# Do Spectra Live in the Matrix? A Brief Tutorial on Applications of Factor Analysis to Resolving Spectral Datasets of Mixtures

**DOI:** 10.1007/s10895-021-02753-w

**Published:** 2021-08-06

**Authors:** Andrzej J. Kałka, Andrzej M. Turek

**Affiliations:** Faculty of Chemistry, Jagiellonian University, 2 Gronostajowa St, 30 387 Cracow, Poland

**Keywords:** Spectral data matrices of mixtures, Excitation-emission maps, Fluorescence quenching, Multivariate curve resolution, Rank annihilation factor analysis, Evolving factor analysis

## Abstract

In spite of a rapid growth of data processing software, that has allowed for a huge advancement in many fields of chemistry, some research issues still remain problematic. A standard example of a troublesome challenge is the analysis of multi-component mixtures. The classical approach to such a problem consists of separating each component from a sample and performing individual measurements. The advent of computers, however, gave rise to a relatively new domain of data processing – chemometry – focused on decomposing signal recorded for the sample rather than the sample itself. Regrettably, still a very few chemometric methods are practically used in everyday laboratory routines. The Authors believe that a brief ‘user-friendly’ guide-like article on several ‘flagship’ algorithms of chemometrics may, at least partly, stimulate an increased interest in the use of these techniques among researchers specializing in many fields of chemistry. In the paper, five different techniques of factor analysis are used for the analysis of a three-component system of fluorophores. These algorithms, applied on the excitation-emission spectra, recorded for the ‘unknown’ mixture, allowed to unambiguously determine its composition without the need for physical separation of the components. An example of using chemometric methods for physical chemistry research is also provided. For each presented technique of the data analysis, a short description of its theoretical background followed by an example of its practical performance is given. In addition, the Reader is supplemented with a basic information on matrix algebra, detailed experimental ‘recipes’, reference specialist literature and ready-to-use MATLAB codes.

**Graphical abstract:**

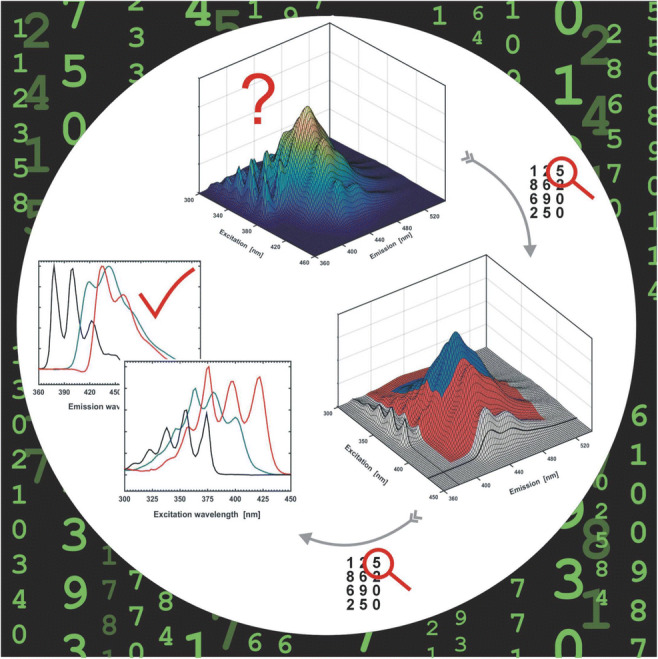

**Supplementary Information:**

The online version contains supplementary material available at 10.1007/s10895-021-02753-w.

## Motivation

Spectroscopic measurements were and still are widely used for determination of both composition and physicochemical properties of the examined samples **[**1**]**. However, interpretation of the obtained spectra, especially in the case of multi-component samples, is not always straightforward. The ‘traditional’ way of obtaining selective signal for each substance, and thus allowing for its unambiguous characterization, is to physically separate it from a mixture **[**2**–**3**]**. This method has, however, a natural limitation, as the separation of all mixture components is not always possible. Often it is also a time consuming procedure.

Hopefully, with the development of computer science, an alternative approach to investigating multicomponent samples has become available. This issue is now addressed by chemometrics. The chemometric techniques combine together chemical knowledge, mathematical and statistical apparatus and numerical optimization routines to effectively extract the desired information out of the data **[**4**–**7**]**. Consequently, there is no need to physically separate components from the mixture. All the required information, concerning the individual signals, is obtained from the computations.

Though there are a plenty of articles in highly specialist literature describing the basics and the usage of the cheomometric techniques, yet still, the application of these methods is rather poorly reflected in the everyday analysis of the complex spectral datasets. Perhaps it is due to the fact that only few of them are explained in a comprehensive way, that is fully understandable for the non-expert audience and illustrated with the help of pictorial presentations [7**–**15].

For this reason, the Authors of this paper attempt to shed an additional light on some of the ‘flagship’ chemometric methods used for resolving spectral mixtures, that are seldom discussed outside the specialist literature. This will include Target Factor Analysis (TFA) [16**–**17], Evolving Factor Analysis (EFA) [18**–**20], Rank Annihilation Factor Analysis (RAFA) [21**–**23] and Generalized Rank Annihilation Method (GRAM) [24**–**25]. Each presented algorithm will be provided with a brief description of its foundations as well as practical details and illustrative examples of its application followed by suggested literature references. Main advantages and some drawbacks will also be discussed.

Four types of supplementary materials have also been included. In **Appendix A****,** the extension of the selected mathematical issues can be found. In **Appendix B****,** the detailed descriptions of the experiments are included, so the measurements can be easily run over. **Appendix C** contains the MATLAB codes [26] for all the applied routines (which may be rewritten in any other freeware programming languages such as R [27] or Python [28]). Finally, in **Appendix D**, the Authors include a set of the originally measured spectral data.

## Theoretical Background

### A Brief Characteristics of UV-vis Spectroscopy

UV-Vis absorption spectroscopy is one of the most commonly used methods for determining the composition or physico-chemical properties of tested samples. As each substance has its ‘unique’ spectrum, UV-Vis measurements can be (and are) used for qualitative analysis purposes. Due to a linear relationship between signal and concentration, UV-Vis spectroscopy is often (and primarily) applied for quantitative analysis. This relationship is described by Lambert-Beer’s law

1a $$ A=\varepsilon \cdotp l\cdotp c $$

where the proportionality factor between absorbance (*A*) and concentration (*c*) is optical path length (*l*) multiplied by molar absorption coefficient (*ε*).

Similar specification of the sample’s composition may also be provided by the UV-Vis emission spectroscopy techniques [29**,** 30]. Then, however, one basic condition must be fulfilled. At least one component of the analysed sample has to reveal fluorescence, phosphorescence or any other type of light emission phenomenon.

Although the Lambert-Beer’s law does not strictly apply to emission spectroscopy, for sufficiently (optically) diluted solutions (absorbance *A* < 0.1) an analogous linear relationship can be obtained. According to Parker’s law [31].

1b $$ {I}_{em}\approx 2.303\cdotp {\varphi}_{em}\cdotp {I}_{source}\cdotp A=2.303\cdotp {\varphi}_{em}\cdotp {I}_{source}\cdotp \varepsilon \cdotp l\cdotp c $$

where the intensity of emitted light (*I*_*em*_, signal) is directly proportional to the concentration of the analyte. Proportionality factors are then, except the already mentioned **(1a)**, the quantum yield of the emission process (*φ*_*em*_) and the intensity of the excitation light beam (*I*_*source*_). Sensitivity of the measurements can be then easily modified by adjusting the parameters of the spectrofluorometer light source.

Although the UV-Vis emission measurements are mostly aimed at delivering the fluorescence or phosphorescence spectra, yet the absorption characteristics of the sample can also be obtained. The fluorescence excitation spectra are then recorded by changing the excitation wavelength and tracking the resulting signal response at one particular emission wavelength. In general, as the emitted light intensity is directly proportional to the absorbance (**1b**), the fluorescence excitation spectra bear a very strong similarity to the absorption spectra.

The combination of the excitation and emission spectra results in the excitation-emission data matrices or maps matrices or (EEM). By changing both the emission and excitation wavelengths during the measurement, it is possible to characterize, at the same time, both the absorption and fluorescent (phosphorescent) properties of the sample.

In some cases, the phenomenon of attenuation of the fluorescence emission intensity is also used. Tiny portions of a substance called the quencher are then added to the sample. The quencher molecules weaken the intensity of the light emitted by fluorophores in the processes including the intermolecular electron or energy transfer between the fluorophore and quencher molecules. Mathematically, this weakening of the fluorescence intensity is described by a linear Stern-Volmer eq. [29**,** 30].

2 $$ \frac{I_{em}^0}{I_{em}^Q}=1+{K}_{SV}\cdotp Q $$

According to the above formula, the emission intensity ratio of the unquenched sample (*I*^*0*^_*em*_) to quenched one (*I*^*Q*^_*em*_) is directly proportional to the concentration of the added quencher (*Q*). The parameter of this proportionality, characteristic for a given pair of a fluorophore and its quencher, is called the Stern-Volmer quenching constant (*K*_*SV*_).

### Spectroscopic Data in Terms of Matrix Algebra

A recorded spectrum, either absorption or emission one, is a set of numerical values representing the intensity of the measured signal (*x*) depending on the wavelength (*λ*). Thus, from a mathematical point of view, the spectrum is a data vector **x** [32].

$$ \mathbf{x}=\left[{x}_1;{x}_2;{x}_3;\dots; {x}_{\lambda}\right] $$

**Fig. 1:**
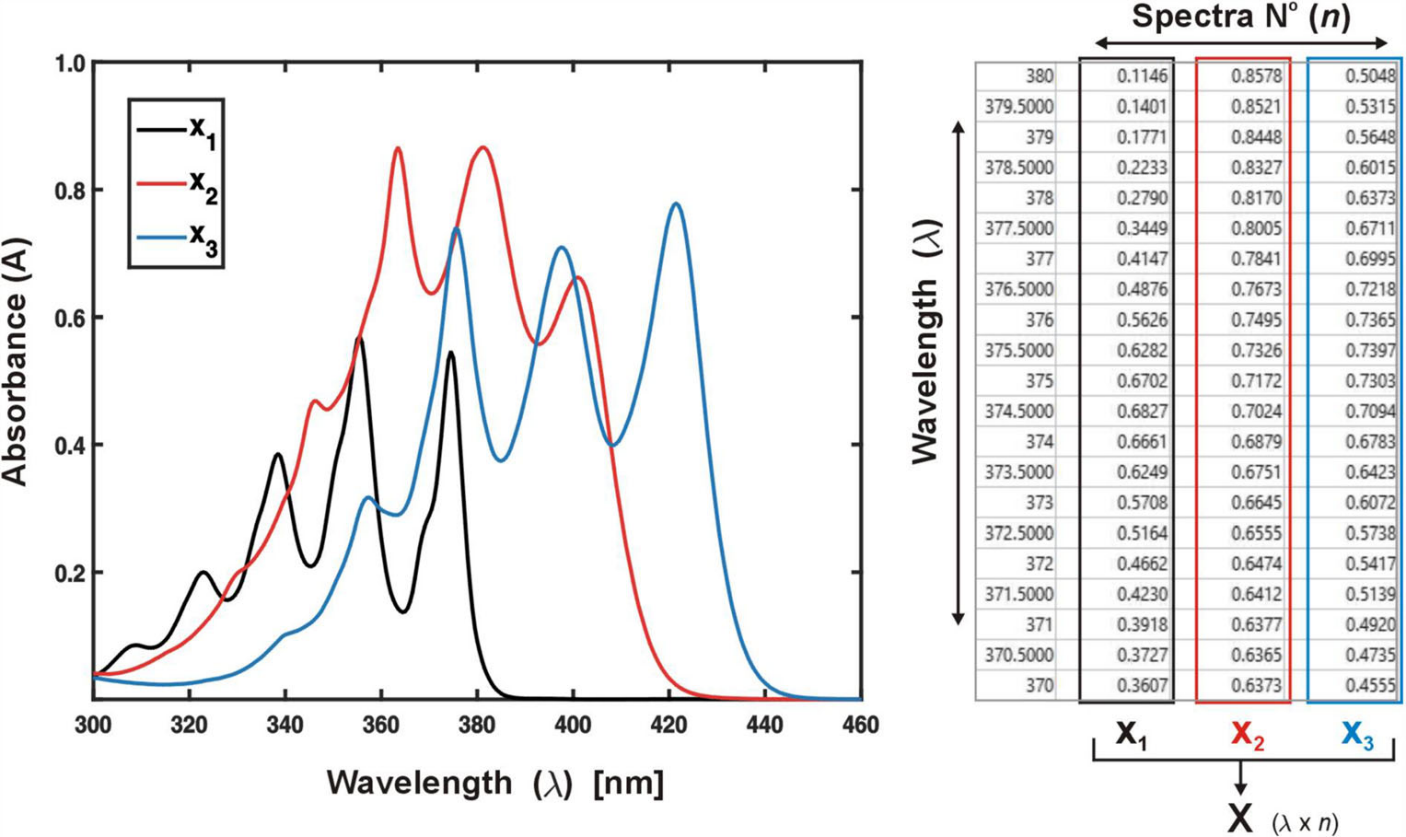
Graphical (left) and matrix representation (right) of spectral data

The vector **x** can be set as a column of values in a data spreadsheet (Fig. 1). Two or more spectra (vectors) combined column-wise form a spectral data matrix **X**:

$$ \mathbf{X}=\left[{\mathbf{x}}_{\mathbf{1}},{\mathbf{x}}_{\mathbf{2}},{\mathbf{x}}_{\mathbf{3}},\dots, {\mathbf{x}}_{\mathbf{n}}\right] $$

The spreadsheet will therefore contain an array with dimensions *λ* x *n*, where *n* - number of combined spectra, *λ* - number of measurement points (set of wavelengths, Fig. 1).

As the recorded signal is directly proportional to concentration (**1a, b**), the spectrum **x**_A_, measured for a particular sample of a substance A, can be expressed as the product of a certain ‘standard’ spectrum **s**_**A**_ related to the unit molar concentration of the solute A and a proper multiplier *c*_A_ representing its actual concentration [5].

$$ {\mathbf{x}}_{\mathbf{A}}={\mathbf{s}}_{\mathbf{A}}\cdotp {c}_A $$

For instance, if three substances, say A, B and C, are mixed together, the resulting spectrum **x**_ABC_ of their three-component mixture, will be, due to signal additivity, a linear combination of three vectors (spectra) representing the individual components.

3 $$ {\mathbf{x}}_{\mathbf{A}\mathbf{BC}}={\mathbf{s}}_{\mathbf{A}}\cdotp {c}_A+{\mathbf{s}}_{\mathbf{B}}\cdotp {c}_B+{\mathbf{s}}_{\mathbf{C}}\cdotp {c}_C $$

By analogy, a set of spectra **x**_**1,ABC**_, **x**_**2,ABC**_, **x**_**3,ABC**_, …, **x**_*n*,ABC_, measured for *n* different mixtures of A, B and C, can be defined as follows

$$ {\displaystyle \begin{array}{c}{\mathbf{x}}_{\mathbf{1},\mathbf{ABC}}={\mathbf{s}}_{\mathbf{A}}\cdotp {c}_{1,A}+{\mathbf{s}}_{\mathbf{B}}\cdotp {c}_{1,B}+{\mathbf{s}}_{\mathbf{C}}\cdotp {c}_{1,C}\\ {}{\mathbf{x}}_{\mathbf{2},\mathbf{ABC}}={\mathbf{s}}_{\mathbf{A}}\cdotp {c}_{2,A}+{\mathbf{s}}_{\mathbf{B}}\cdotp {c}_{2,B}+{\mathbf{s}}_{\mathbf{C}}\cdotp {c}_{2,C}\\ {}\dots \\ {}{\mathbf{x}}_{\mathbf{n},\mathbf{ABC}}={\mathbf{s}}_{\mathbf{A}}\cdotp {c}_{n,A}+{\mathbf{s}}_{\mathbf{B}}\cdotp {c}_{n,B}+{\mathbf{s}}_{\mathbf{C}}\cdotp {c}_{n,C}\end{array}} $$

The above set of equations can be rewritten briefly in matrix notation as

4 $$ \mathbf{X}=\mathbf{S}\cdotp {\mathbf{C}}^{\mathbf{T}} $$

**Fig. 2:**
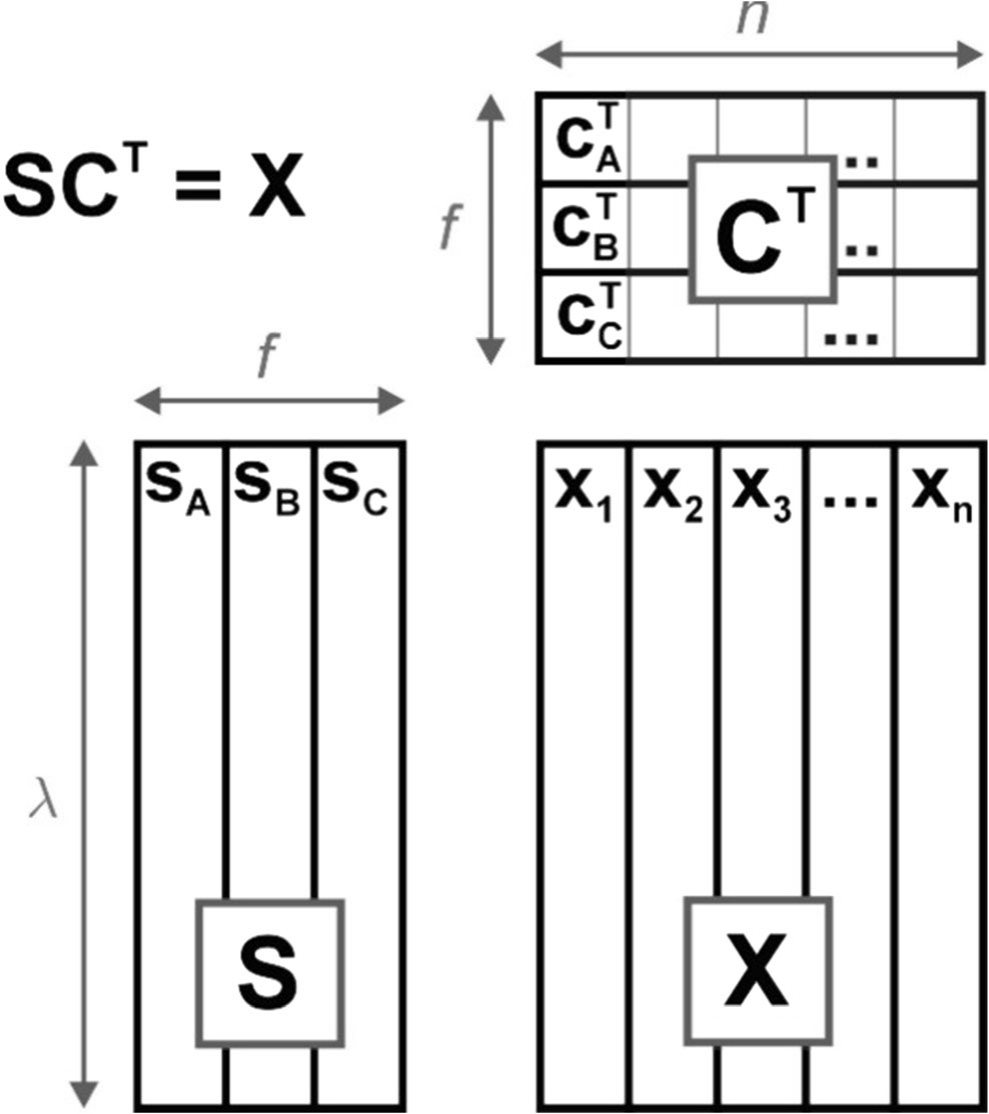
Scheme showing matrix formulation of Lambert-Beer law. The data matrix **X**, containing n spectra of a three-component mixture, is decomposed into the product of matrices **S** and **C**, respectively, consisting of individual spectral and concentration (intensity) profiles of all components

By general consent, the matrix **S**, called a matrix of *f* (in this example it equals 3) spectral profiles, contains the standard spectra **s**_**A**_, **s**_**B**_ and **s**_**C**_ of ‘pure’ substances A, B and C. The vectors **c**_A_, **c**_B_ and **c**_C_ representing the actual concentrations of components A, B and C are columns of the matrix **C** sized *n* × 3, called a matrix of concentration profiles. Symbol T denotes the operation of matrix transposition. A graphical scheme illustrating the described matrix factorization is presented in Fig. 2.

Hence, having a set of ‘standard’ spectra of all components of a mixture, the concentrations of all substances (A, B and C) in each sample can be determined by performing a simple matrix operation:

5a $$ {\mathbf{C}}^{\mathbf{T}}={\mathbf{S}}^{+}\mathbf{X} $$

The symbol **S**^**+**^ denotes a matrix pseudo-inverse

$$ {\mathbf{S}}^{+}={\left({\mathbf{S}}^{\mathbf{T}}\mathbf{S}\right)}^{-\mathbf{1}}{\mathbf{S}}^{\mathbf{T}} $$

with the property **S**^**+**^**S = 1**, obtained upon ‘inversion’ of a rectangular matrix [33] (as shown in **SI - App. A.1**).

### Singular Value Decomposition of a Data Matrix and its ‘Consequences’

**Fig. 3:**
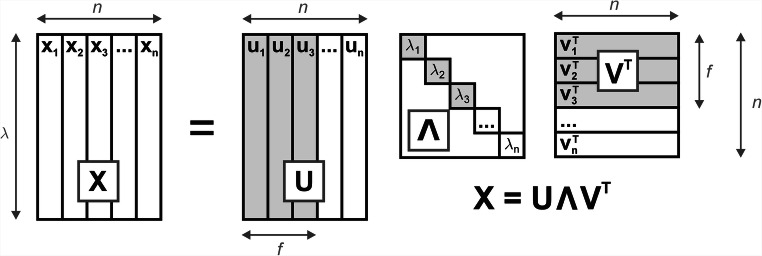
Scheme showing the decomposition of data matrix **X** from Fig. 2 into the product of three matrices **U**, **Λ** and **V**^**T**^ with the SVD algorithm. Submatrices, to which these matrices may be reduced for the purpose of data reproduction are marked in grey

In everyday laboratory practice, it oftentimes happens that both matrices containing the spectral (**S**) and concentration (**C**) profiles of individual components remain unknown, so the **X** matrix decomposition given by formula **(****4****)** cannot be directly used. However, by applying a mathematical procedure known as Singular Value Decomposition (SVD), it is always possible to decompose the data matrix **X** into a product of three matrices, by convention usually denoted as **U**, **Λ** and **V** (Fig. 3) [5].

6 $$ \mathbf{X}=\mathbf{U}\boldsymbol{\Lambda } {\mathbf{V}}^{\mathbf{T}} $$

The SVD matrices **U** (*λ* x *n*) and **V** (*n* x *n*), consisting of two sets of eigenvectors, are characteristically structured with the property of column-wise orthonormality(**U**^**T**^**U** = **1** and **V**^**T**^**V** = **1**, and in addition **VV**^**T**^ **= 1**) [5**,** 34]. Matrix **Λ** (*n* x *n*) is a diagonal matrix containing the singular values of the matrix **X**.

**Fig. 4:**
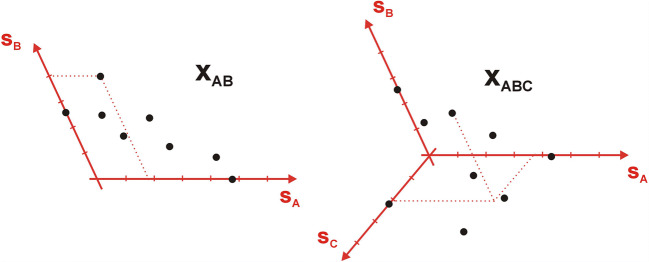
Geometric interpretation of two- and three-component **x**_**AB**_ and **x**_**ABC**_ mixture spectra. All spectra (black dots) are represented by points located in the coordinate system defined by the ‘standard’ spectra of ‘pure’ components **s**_**A,**_ **s**_**B**_ and **s**_**C**_. The coordinates (dashed lines) are identical to the scaling factors (concentrations) c_A_, c_B_ and c_C_

To understand the meaning and importance of the decomposition of the data matrix **X** into a product of three matrices, which actually do not contain the spectra or concentrations of pure components, a visual reference to geometry may be made (Fig. 4). The formula **(****3****)** defining a spectrum of the mixture **x**_**ABC**_ as the sum of the individual components spectra can be seen as analogous to the space representation of a certain vector **p** in the Cartesian coordinate system [32].

$$ {\displaystyle \begin{array}{c}\mathbf{p}=x\cdotp \mathbf{x}+y\cdotp \mathbf{y}+z\cdotp \mathbf{z}\\ {}p=\left(x,y,z\right)\end{array}} $$

The versors **x**, **y** and **z** are then identical to the vectors representing the ‘pure’ components spectra **s**_**A**_, **s**_**B**_ and **s**_**C**_. The multipliers (concentrations) *c*_*A*_, *c*_*B*_ and *c*_*C*_ stand for the respective ‘coordinates’. The axes of such a coordinate system, in general, do not have to be mutually orthogonal [34].

Consequently, if the spectra of ‘pure’ components are unknown, the problem arises how to define the axes of such a coordinate system, that would allow to describe all the collected mixture spectra. And this is just when the SVD procedure comes to the aid. One can find a set of potentially useful axes (Fig. 4) in the matrix **U**. However, as this matrix contains up to *n* eigenvectors **u (**Fig. 3), the decision has to be made how many and which of them should be chosen.

**Fig. 5:**
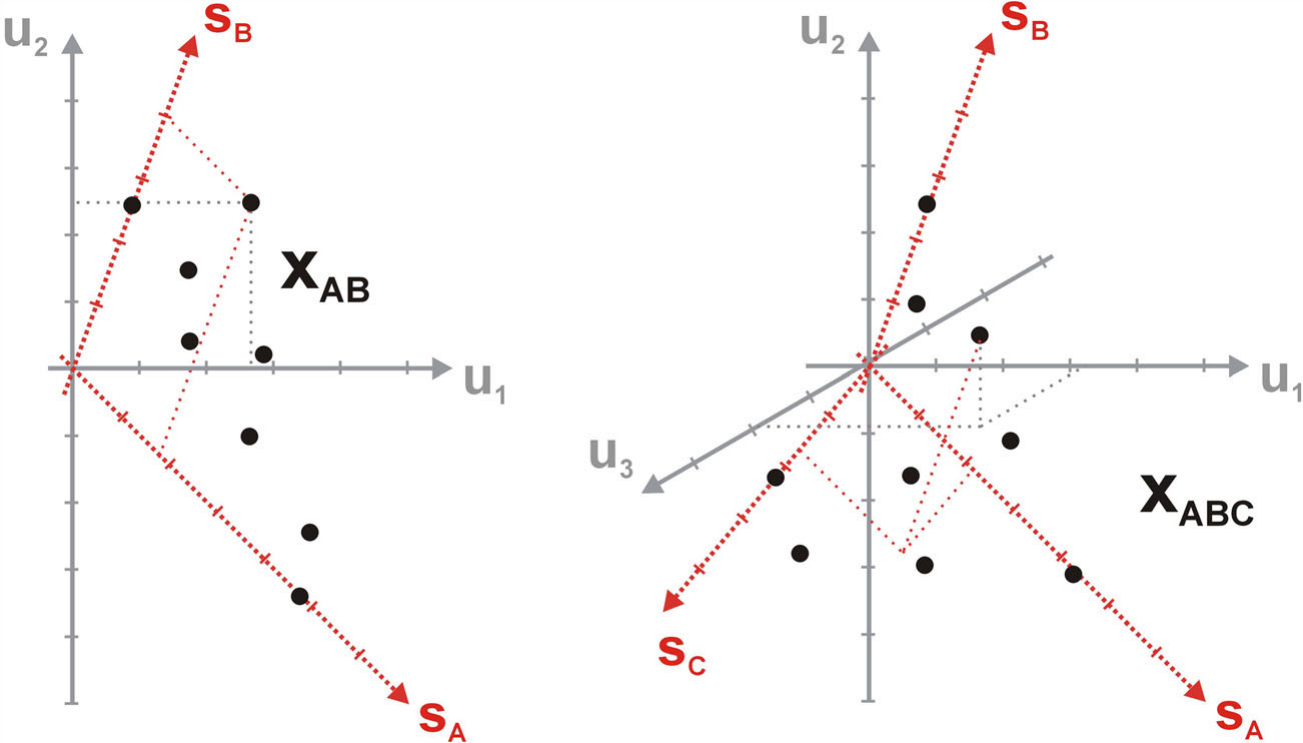
Geometric interpretation of eigenvectors **u**, obtained by SVD of the data matrix **X**. An appropriate set of such orthogonal vectors allows to draw a coordinate system describing the experimental data points. This is particularly useful when the spectra **s**_**A**_, **s**_**B**_ and **s**_**C**_ of pure’ components, and hence the ‘original’ axes of the system remain unknown (cf. Figure 4 - the ‘red’ remains the same, but have been rotated)

Information on how many axes are actually needed to describe the measurement data matrix **X** and hence how many components are present in the mixture, can actually be gleaned from the diagonal matrix of singular values **Λ**. From the point of view of linear algebra, the recommended dataset consists of as many independent variables (geometrically – axes) as is the determined number of singular values which are distinctively greater than zero [5]. It is therefore possible to ‘truncate’ the **U**, **Λ** and **V** matrices into the ‘proper’ number of *f* columns (Fig. 3**-** grey areas). The ‘truncation’ is commonly marked with a bar above a ‘reduced’ quantity. The cut-off number *f* is called the number of significant factors, principal components or primary latent variables. A ‘recipe’ for drawing the desired coordinate system of **X** dataset is thus finally obtained. Although, in general, the set **Ū** of orthogonal axes defined in this way will not overlap with the ‘original’ axes, corresponding to the ‘pure’ component spectra **s**_**A**_, **s**_**B**_ and **s**_**C**_, the space spanned by the vectors **u**_**1**_, **u**_**2**_ and **u**_**3**_ will remain identical (Fig. 5):

$$ {\mathbf{u}}_{\mathbf{1}}\cdotp {\lambda}_1\cdotp {v}_{n,1}+{\mathbf{u}}_{\mathbf{2}}\cdotp {\lambda}_2\cdotp {v}_{n,2}+{\mathbf{u}}_{\mathbf{3}}\cdotp {\lambda}_3\cdotp {v}_{n,3}={\mathbf{x}}_{\mathbf{n},\mathbf{ABC}}={\mathbf{s}}_{\mathbf{A}}\cdotp {c}_{n,A}+{\mathbf{s}}_{\mathbf{B}}\cdotp {c}_{n,B}+{\mathbf{s}}_{\mathbf{C}}\cdotp {c}_{n,C} $$

It is therefore quite easy to notice (see **SI - App. A.3**) that the vectors **u**_**1**_, **u**_**2**_ and **u**_**3**_ are linear combinations of the pure component spectra **s**_**A**_, **s**_**B**_ and **s**_**C**_.

7a $$ {\overline {\mathbf{u}}}_{\mathbf{i}}={r}_{i,A}\cdotp {\mathbf{s}}_{\mathbf{A}}+{r}_{i,B}\cdotp {\mathbf{s}}_{\mathbf{B}}+{r}_{i,C}\cdotp {\mathbf{s}}_{\mathbf{C}} $$

Needless to say this relationship is reflexive

7b $$ {\mathbf{s}}_{\mathbf{i}}={r}_{i,1}^{\prime}\cdotp {\mathbf{u}}_{\mathbf{1}}+{r}_{i,2}^{\prime}\cdotp {\mathbf{u}}_{\mathbf{2}}+{r}_{i,3}^{\prime}\cdotp {\mathbf{u}}_{\mathbf{3}} $$

and can be rewritten in a concise matrix notation as

7c $$ {\overline {\mathbf{u}}}_{\mathbf{i}}=\mathbf{S}{\mathbf{r}}_{\mathbf{i}}\kern1.33em \mathrm{and}\kern1.33em {\mathbf{s}}_{\mathbf{i}}=\overline {\mathbf{U}}{\mathbf{r}}_{\mathbf{i}}^{\prime } $$

Of course, the set of linear combination coefficients **r** and **r’** remains unknown until the true spectra **S** are recovered. Nevertheless, the properties of the SVD matrices presented above are very useful in the analysis of the complex spectroscopic data.

Finally, the procedure of data reproduction is also worth mentioning. It consists of calculating the product of the **U**, **Λ** and **V** matrices, ‘truncated’ to *f* columns (Fig. 3)

8 $$ \mathbf{X}=\overline {\mathbf{X}}+\mathbf{E}=\overline {\mathbf{U}}\overline {\boldsymbol{\Lambda}}\overline {{\mathbf{V}}^{\mathbf{T}}}\kern0.45em +\mathbf{E} $$

As a result, the original dataset in the **X** matrix is ‘idealised’ to the *f*-variate system. Any imperfections, that do not fit into the adopted *f*-component model, are rejected. These ‘misfits’, collected in the matrix **E**, known as the error matrix, are often assumed to represent the undesirable measurement noise [5].

## Experimental Model System

**Fig. 6:**
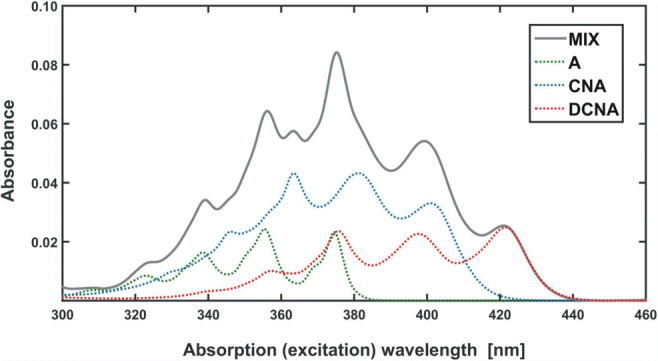
Absorption spectrum of a model three-component mixture (MIX) with marked contributions of all components (A, CNA, DCNA)

To present a practical use of the factor analysis apparatus for interpretation of spectroscopic data, a model experimental system was prepared (see **SI - App. B**). Methanol solutions of anthracene (A), 9-cyanoanthracene (CNA), 9,10-dicyanoanthracene (DCNA) and 9,10-diphenylanthracene (DPhA) were chosen for the study [35**–**36]. This choice was motivated by the fact, that anthracene and its derivatives show an easy to measure fluorescence phenomenon. In addition, the selected substances can mimic a post-reaction mixture, hypothetically obtained in the synthesis of monocyano derivative (CNA) from anthracene (A). Dicyano derivative (DCNA) is then a by-product and DPhA can be treated as an impurity that should not be present in the reaction system. Thus, a three-component mixture of A, CNA and DCNA was prepared with a proportion of 0.4 cm^3^, 0.5 cm^3^ and 0.3 cm^3^ of base solutions (see **SI - App. B,** Fig. 6). In order to maintain the linear dependence of the signal on concentration **(1b)**, the proper dilution of all the solutions was kept. The controlled maximum absorbance was always lower than the limit value of 0.1 (i.e. in Fig. 6) [31].

**Fig. 7:**
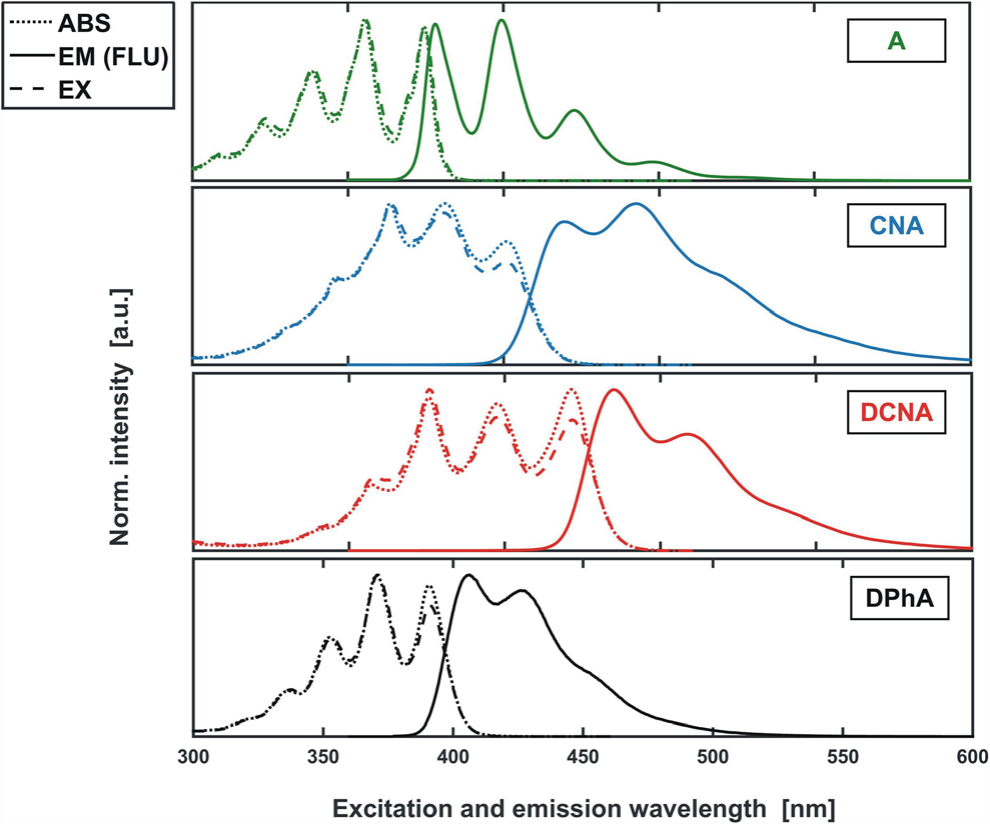
Fluorescence (continuous line), absorption (dotted line) and excitation (dashed line) spectra of the studied fluorophores normalised to a unit maximum

For each fluorophore, as well as for the mixture, the set of absorption, excitation (EX) and emission (EM) spectra was measured (Fig. 7). For the CNA and DCNA samples the excitation-emission maps (EEM) were also recorded.

## A Practical Example of Factor Analysis Performed on Excitation-Emission Maps

### How Many Components Are in a Mixture?

**Fig. 8:**
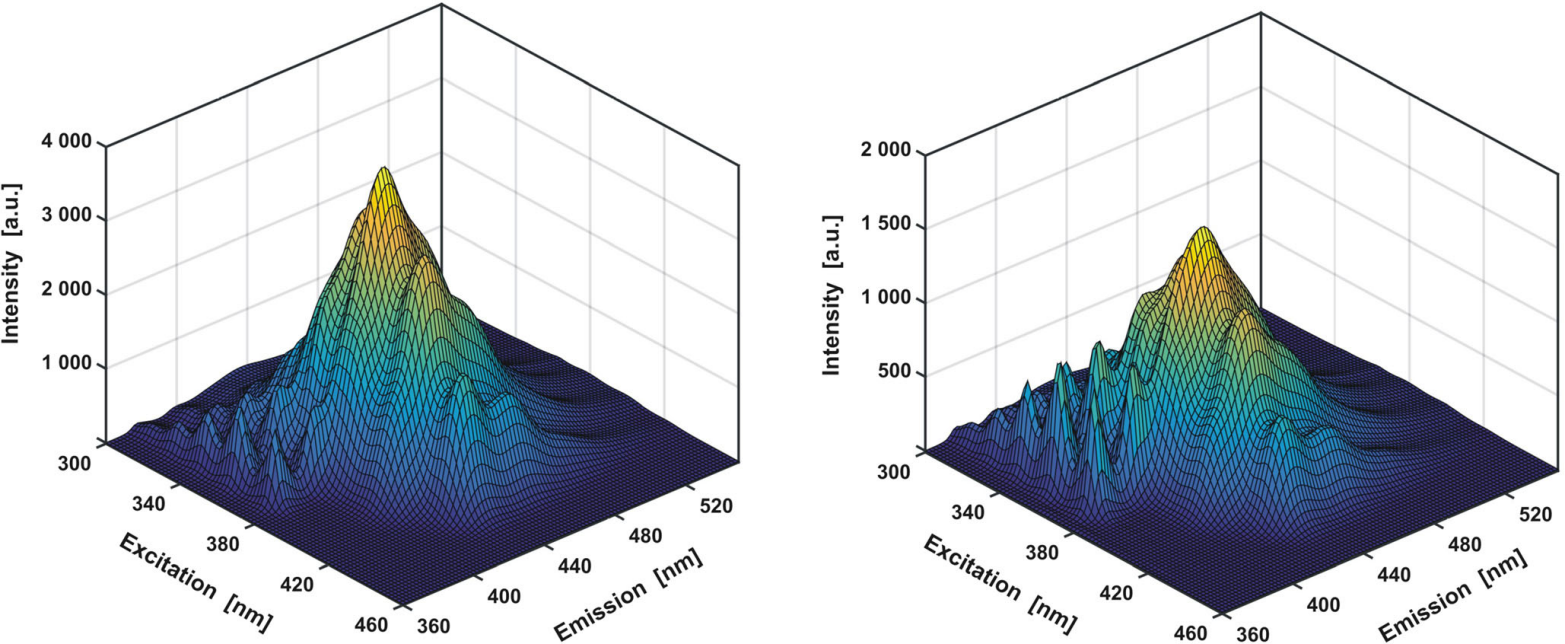
Excitation-emission map recorded for a mixture of three fluorophores before (left) and after addition of potassium iodide as a quencher (right). In the emmision range of 300–380 nm, characteristic, protruding ‘sharp’ bands (originating from anthracene) can be observed

By looking at a single absorption or emission spectrum of the ‘unknown’ mixture (Fig. 6), it is usually very difficult to determine how many components it consists of. However, the ‘pack’ of several spectra grouped in a form of an excitation-emission map (EEM), seems to be much more informative. Some ‘extra’ knowledge may be also revealed when a quencher is added to the sample (Fig. 8), as intensity of each fluorescent species is quenched at a slightly different rate **(2)**.

In the studied case, even a ‘quick look’ at the recorded EEM reveals that the spectra could be divided into (at least) two distinct categories (Fig. 8). The first is characterised by a set of ‘spiky’ bands while the other is predominated by ‘smooth’ and ‘diffused’ bands. This distinction becomes even more apparent upon the addition of potassium iodide (KI) as a quencher (Fig. 8 – right panel, **SI** – Appendix **B.4.3**). Therefore, it can be immediately stated that the mixture consists of at least two components. However, in order to determine the correct number of significant factors responsible for the total variance of the analysed dataset, a more sophisticated and reliable method than ‘organoleptic’ assessment should be employed. Principal Component Analysis (PCA) is one of the most popular approaches suitable for that purpose [37]. As PCA was already widely discussed elsewhere (for relevant examples see [8**,** 13]), only the main features will be prompted below.

Since the excitation-emission map can be treated as a data matrix **X**_**MIX**_, it can be factorized with SVD. A set of singular values **Λ** is then obtained **(6)**. Just a reminder, the number of large non-zero singular values *λ* (or eigenvalues) should be equal to the number of significant factors responsible for the variance of the analysed dataset. In order to distinguish between significant and zero-like singular values [5**,** 13], some statistical criteria as those proposed by Malinowski [38] **(S.5, S.6)** can be additionally applied (see Table 1 and **SI - App. A.4**).

**Table 1:** Subsequent f singular values λ of the **X**_**MIX**_ data matrix (Fig. 8), consisting of 81 fluorescence spectra, with the corresponding parameters of relative σ^2^ **(S. 5)** and summaric explained variance Σ **(S. 6)**. The indicated number of significant factors (f = 3) is marked with an exclamation mark (**!**)

–	*f* = 1	*f* = 2	*f* = 3 (**!**)	*f* = 4	*f* = 5	*f* = *n* = 81
Excitation-emission map without the quencher						
*λ value*	78,436	9158	3975	167	163	0.998
*σ*^*2*^ *rel.var.*	98.4%	1.34%	0.253%	4.46·10^−4^%	4.23·10^−4^%	4,59·10^−8^%
*Σ sum. Var.*	98.40%	99.74%	99.995%	99.995%	99.996%	100.0%
Excitation-emission map with the added quencher						
*λ value*	33,726	6552	1736	187	135	1.08
*σ*^*2*^ *rel.var.*	96.1%	3.63%	0.254%	2.95·10^−4^%	1.53·10^−4^%	9,83·10^−8^%
*Σ sum. Var.*	96.10%	99.73%	99.981%	99.984%	99.986%	100.0%

Complementary, a graphical analysis of the eigenvectors can also be performed [39]. As significant eigenvectors and ‘pure’ component spectra are mutually related (**7a-c,** Fig. 5), the ‘shape’ of a significant eigenvector should somehow resemble the shape of the measured UV-Vis spectra (wide and diffused ‘bands’). On the other hand, all non-significant eigenvectors are expected to have an irregular, chaotic shape, representing the random incidental noise [39**,** 5].

**Fig. 9:**
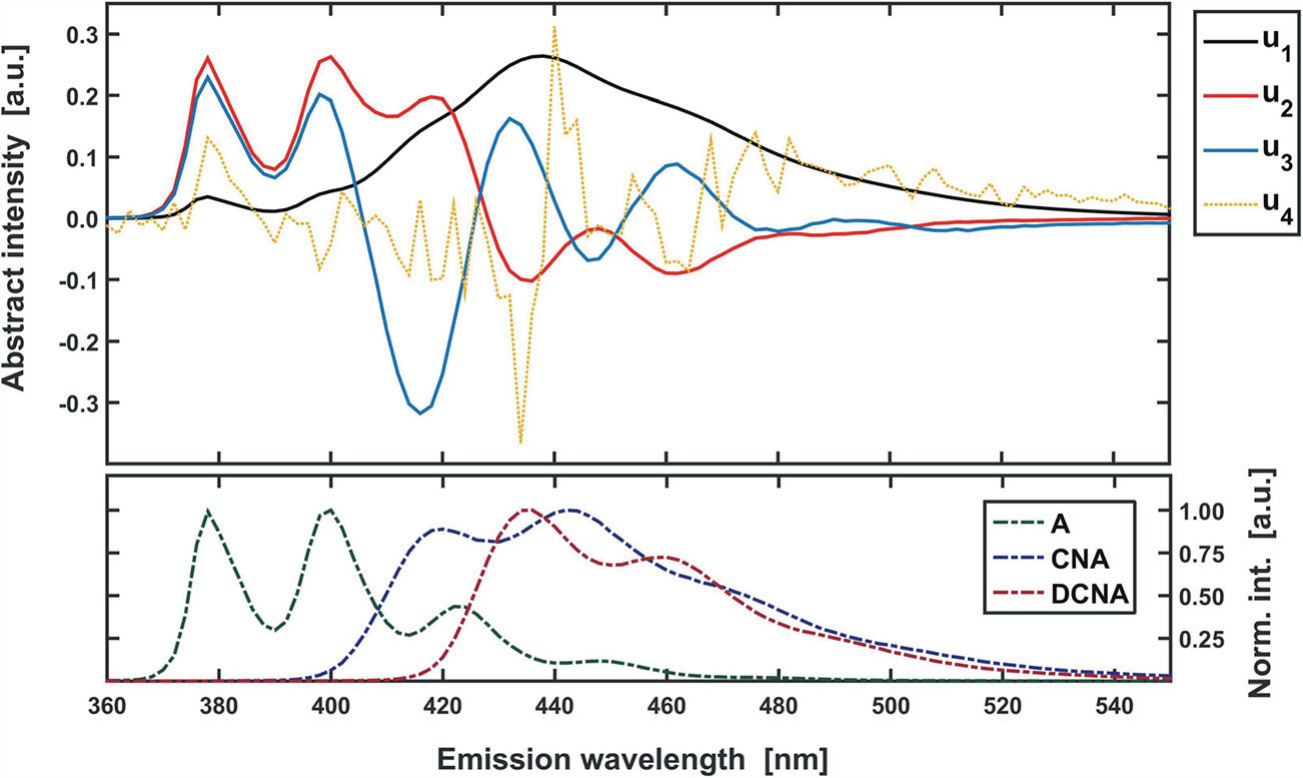
Top: first four eigenvectors **u** obtained for dataset **X**_**MIX**_ (Fig. 8**– left panel**). The first three of them are characterized by a regular pattern, while the fourth one reflects a random, chaotic noise. At the bottom: the fluorescence spectra of the three mixture components (Fig. 7). A correlation can be seen between the abstract (top) and real (bottom) spectra (i.e. in extreme positions – see **SI – App. A.3**)

By looking at the subsequent eigenvectors **u** of the matrix **X**_**MIX**_ (Fig. 9), it can be noticed that only first three of them have a ‘regular’ shape. The fourth eigenvector (and all that follow) remain ‘rugged’ and do not exhibit any characteristic features. It can be therefore concluded, that the full excitation-emission map is made up of combinations of only three independent spectra, which is fully consistent with the true composition of the analysed three-component sample (A + CNA + DCNA).

In general, on the basis of the applied criteria the number of fluorescent components in a mixture can be reliably determined (for the PCA routine –see **SI**, App. **C.1**). Yet, it is still unclear what these substances are or what their concentration is. The obtained results tend to prove that the computational analysis of the spectra may successfully replace ‘traditional’ methods, such as chromatography [2] or electrophoresis [3], which, at this point, could allow obtaining a similar outcome.

### Which Substances May Be or Be Not Present in a Studied Mixture?

If the analysed sample is suspected to contain some known substances, the SVD of the excitation-emission data matrix may be used to confirm or reject this presumption. The Target Factor Analysis (TFA) approach is specifically dedicated for that purpose [16**–**17**,** 9]. The first step of TFA is to estimate a limited set of substances potentially present in a sample. Then, the adequate spectra of all these substances obtained either from personal measurements and/or a proper spectral database should be gathered. Next, a following reasoning may be carried out. If the mixture actually contains one of the ‘targeted’ substances, its spectrum should be related to the abstract spectra of the analysed data matrix by a linear transformation (**7a-c**, Fig. 5). It means that a proper combination of the significant eigenvectors **u** is expected to fully reproduce the ‘target’ test spectrum **s**_**T**_ **(7b)**. At the same time, if the substance was not present in the analysed sample, then in general, neither combination of abstract spectra **u** will be able to fully restore its ‘original’ spectrum.

The mathematical formulation of the above conclusion can be performed in three consecutive steps. Firstly, for the ‘target’ spectrum **s**_**T**_, the optimum coefficients **r** of a linear combination of the significant eigenvectors **u** are determined **(7a)**.

$$ \mathbf{r}={\overline {\mathbf{U}}}^{+}{\mathbf{s}}_{\mathbf{T}} $$

Then, on the basis of the calculated **r** values, a ‘new’ spectrum **ŝ**_**T**_ is reconstructed from the eigenvectors **(7b)**.

$$ {\hat{\mathbf{s}}}_{\mathbf{T}}=\overline {\mathbf{U}}\mathbf{r} $$

Finally, the reproduced spectrum **ŝ**_**T**_ is compared to the ‘initial’ one, **s**_**T**_.

$$ \operatorname{}{\mathbf{s}}_{\mathbf{T}}={\hat{\mathbf{s}}}_{\mathbf{T}}\mid {\mathbf{s}}_{\mathbf{T}}\ne {\hat{\mathbf{s}}}_{\mathbf{T}} $$

Equivalently, it can be said that the ‘target’ test spectrum **s**_**T**_ is projected on the set of the significant eigenvectors, defining the dimensions of the predicted data-points space (Fig. 5, see **SI - App. A.3**). The projection product **ŝ**_**T**_ is then compared with the ‘original’ target spectrum **s**_**T**_.

**Fig. 10:**
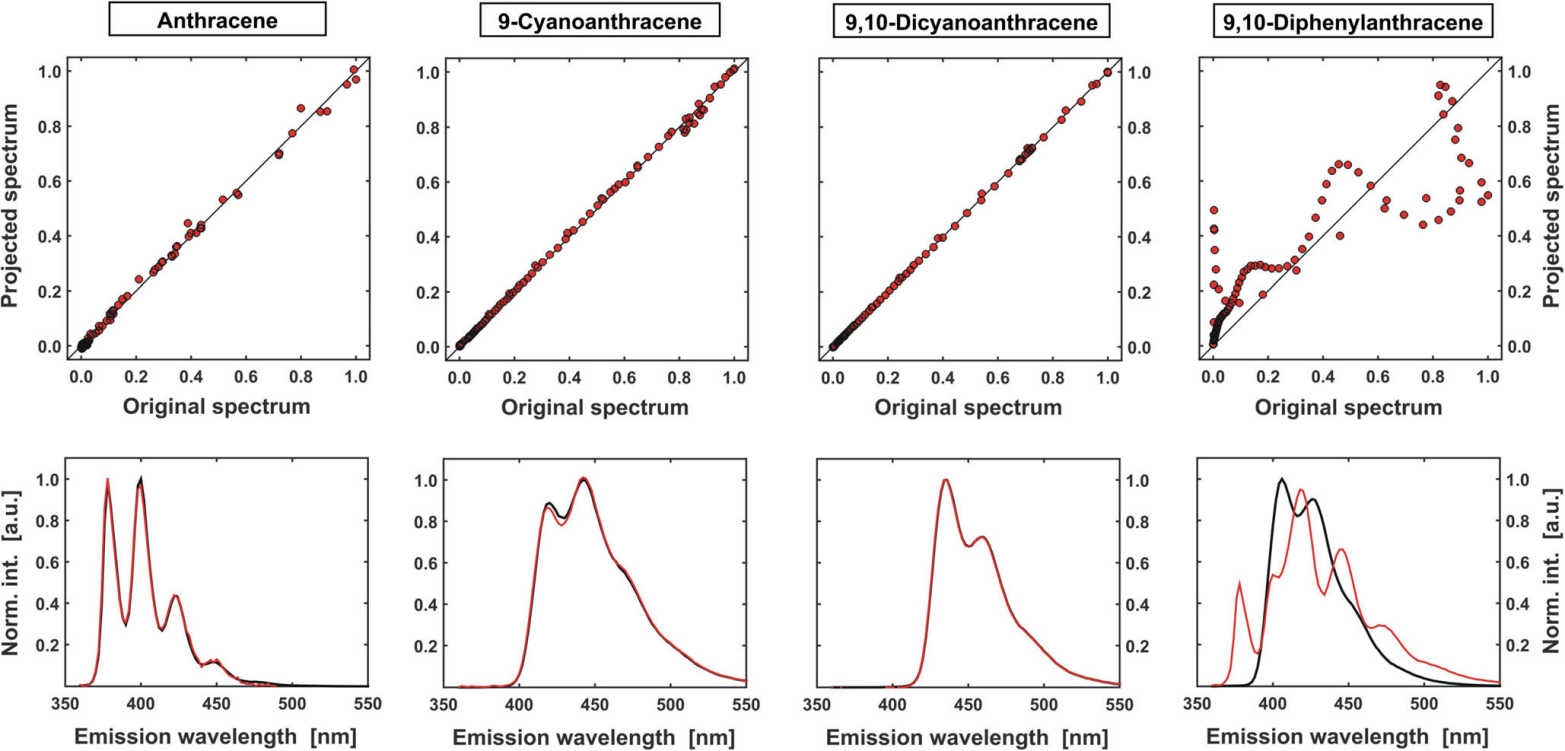
Schematic diagram of the TFA procedure carried out for an ‘unknown’ sample and spectra of four ‘target’ substances: A, CNA, DCNA and DPhA (black lines). The first three of them are well reproduced by a combination of eigenvectors (red lines), which seems to confirm their presence in a mixture. For the spectrum of DPhA, this regularity does not exist, which indicates that it was not a component of the sample

The comparison between the two vectors can be done graphically. Values of the subsequent elements of **s**_**T**_ are then put on the x-axis and the corresponding values of **ŝ**_**T**_ are placed on the y-axis are plotted against them (Fig. 10). If the ‘target’ test spectrum indeed had a contribution to the measured spectra of the analysed mixture, then both **s**_**T**_ and **ŝ**_**T**_ spectra will be almost identical. A one-to-one correlation (a straight line y = x) will then be observed. However, if the original and projected spectrum remain significantly different (the linear correlation is no longer preserved), it can be concluded, that the ‘targeted’ substance was not a component of the sample.

The above algorithm (see TFA routine – **SI**, App. **C.3**) was applied on the model excitation-emission map **X**_**MIX**_ and a set of the individual fluorescence spectra of A, CNA, DCNA and DPhA (Fig. 7) used as ‘targets’ (see SI – Appendix B.4.2). A linear correlation in the plots (Fig. 10) is observed for the first three of them, which suggests that the mixture consists of A, CNA and DCNA. On the other hand, the ‘original’ spectrum of DPhA and the spectrum ‘assembled’ from eigenvectors **u** remain significantly different. The absence of DPhA in the sample is thus graphically confirmed.

On the basis of the presented example, the target factor analysis can be seen as the powerful tool to validate the composition of an analysed sample, provided that some auxiliary adequate ‘targets’ are available. Consequently, TFA should be of great interest especially in synthetic chemistry, as it allows to assess a purity of the final products in view of the presence of possible contaminations.

### How Much of a Component Is in a Sample?

Factor analysis allows also to determine the amount of a given substance in a sample, without a need of its physical separation. One of the algorithms dedicated for this purpose is the Rank Annihilation Factor Analysis (RAFA) [21**–**23]. If the adequately ‘calibrated’ spectra, **S**, of all components of a mixture are known, then the simultaneous determination of all the component concentrations, **C,** may be performed by the already mentioned direct matrix calculation **(5a)**

$$ {\mathbf{C}}^{\mathbf{T}}={\mathbf{S}}^{+}{\mathbf{X}}_{\mathbf{MIX}} $$

But what if the researcher is interested in determining the concentration of only few selected components, i.e. the main products of the synthesis, or a given type of contamination? As an alternative to preparing a series of calibration solutions for all the mixture components (also for those, that are not under consideration), the following reasoning can be performed. Since in the UV-Vis measurements the signals are additive, the spectra of a mixture can be presented as the sum of the spectra of individual components. The excitation-emission map recorded for the mixture of A, CNA and DCNA, **X**_**MIX**_, would be then a sum of three matrices

$$ {\mathbf{X}}_{\mathbf{MIX}}={\mathbf{X}}_{\mathbf{A}}+{\mathbf{X}}_{\mathbf{CNA}}+{\mathbf{X}}_{\mathbf{DCNA}} $$

combining the contributions of particular components.

Analogically, by measuring the excitation-emission map for a calibration sample of an individual component, i.e. CNA, a reference EEM matrix, **Y**_**CNA**_, is obtained. Because the signal remains directly proportional to the concentration ***(1a, b)****,* for any pair of the corresponding entries of the **X**_**CNA**_ and **Y**_**CNA**_ matrices, the following relation is fulfilled.

11 $$ \frac{x_{CNA}}{y_{CNA}}=\frac{c_x}{c_y}={\tau}_0 $$

The searched, unknown concentration of CNA in the analysed sample is denoted by *c*_*x*_, while the well determined concentration of the standard by *c*_*y*_. In the matrix notation, the above can be written as

$$ {\mathbf{X}}_{\mathbf{CNA}}=\frac{c_x}{c_y}\cdotp {\mathbf{Y}}_{\mathbf{CNA}}={\tau}_0\cdotp {\mathbf{Y}}_{\mathbf{CNA}} $$

The scaling parameter *τ*_*0*_ is here the ratio of the CNA concentration in the analysed and reference (calibration) sample. Consequently, the **X**_**MIX**_ matrix can be presented as:

$$ {\mathbf{X}}_{\mathbf{MIX}}={\mathbf{X}}_{\mathbf{A}}+{\tau}_0\cdotp {\mathbf{Y}}_{\mathbf{CNA}}+{\mathbf{X}}_{\mathbf{DCNA}} $$

Of course, the value of *τ*_*0*_ remains unknown as is *c*_*x*_. However, it can easily be determined by the following scheme. Let the reference **Y**_**CNA**_ matrix, scaled by any *τ* parameter, be subtracted from **X**_**MIX**_. A resulting difference matrix **D**_**MIX**_ will be then produced.

12 $$ {\mathbf{D}}_{\mathbf{MIX}}={\mathbf{X}}_{\mathbf{MIX}}-\tau \cdotp {\mathbf{Y}}_{\mathbf{CNA}}={\mathbf{X}}_{\mathbf{A}}+\left({\tau}_0-\tau \right)\cdotp {\mathbf{Y}}_{\mathbf{CNA}}+{\mathbf{X}}_{\mathbf{D}\mathbf{CNA}} $$

In general, the number of significant factors determined for the matrix **D**_**MIX**_ will be three (*f* = 3), as was in the case of the data matrix **X**_**MIX**_. However, if the value of the arbitrarily adopted parameter *τ* is coincidentally equal to *τ*_*0*_, then the difference matrix **D**^**0**^_**MIX**_ will consist only of two components:

$$ {\mathbf{D}}_{\mathbf{MIX}}^{\mathbf{0}}={\mathbf{X}}_{\mathbf{MIX}}-{\tau}_0\cdotp {\mathbf{Y}}_{\mathbf{CNA}}={\mathbf{X}}_{\mathbf{A}}+{\mathbf{X}}_{\mathbf{D}\mathbf{CNA}} $$

as the contribution of CNA will be annihilated. As a result, the number of significant non-zero singular values of **D**_**MIX**_ will be reduced by one (from three to two). The ‘last’ significant singular value *λ*_*f*_ (in the studied case – the third one) will be, then, a kind of an ‘indicator’, that can be used to find the ‘correct’ value of *τ*. As *τ* ‘approaches’ *τ*_*0*_, the value of *λ*_*f*_ decreases and at ‘critical point’ (*τ* = *τ*_*0*_), it will reach a value close to zero. Although a random search for the optimal *τ* value is always possible, a definitely more efficient approach is to launch a systematic search. A sequence of scaling parameters τ is then produced (i.e. *τ* = 0.00, 0.01, 0.02, ..., 1.00) and the evolution of the *f*-th singular value of **D**_**MIX**_ is traced. This is the so called iterative variant of rank annihilation factor analysis [21**,** 22]. An alternative, direct version [23] of this approach will be discussed in **Chapter 4.5** (GRAM).

**Fig. 11:**
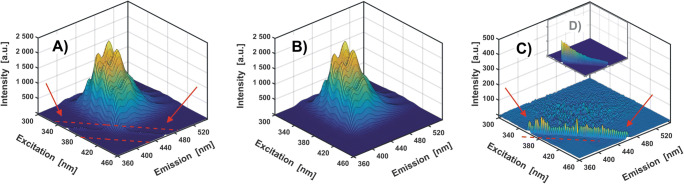
Excitation-emission map of CNA calibration sample before (A) and after (B) reproduction. On the difference map (C) a ‘rugged’ structure, representing instrumental noise, and Rayleigh scattering band residuals (marked with red lines and arrows) are observed. For comparison, a miniature of EEM of the pure solvent (methanol) is shown (D)

In the case of a model mixture of three fluorophores discussed here, an exemplary quantitative RAFA procedure (RAFA routine – see **SI**, App. **C.4**) will consist in determining the amount of CNA acting as the main reaction product. The excitation-emission maps for the calibration sample (0.5 cm^3^ / 10 cm^3^, see **SI - App. B**) have to be then recorded (Fig. 11). For comparison, the DCNA contribution, corresponding to the by-product, will also be quantified.

At this point, it is worth to briefly describe the method of ‘idealising’ the measured data by their reproduction based on SVD (see routine **C.2** in **SI**, App. C). The analysis of the SVD matrices obtained for the **Y**_**CNA**_ matrix (**Chapter 4.1**) yields one significant singular value *λ* and one pair of vectors **u** and **v**^**T**^ (only one variable - CNA). By reproducing the excitation-emission matrix of CNA as **(8)**

$$ {\overline {\mathbf{Y}}}_{\mathbf{CNA}}={\mathbf{u}}_{\mathbf{1}}{\lambda}_1{\mathbf{v}}_{\mathbf{1}}^{\mathbf{T}} $$

a noticeable ‘improvement’ in the shape of EEM can be observed (Fig. 11). Compared to the ‘raw’ data, the random noise and residues from the Rayleigh scattering band, which are a characteristic obstacle for the analysis of the excitation-emission maps, are successfully removed.

**Fig. 12:**
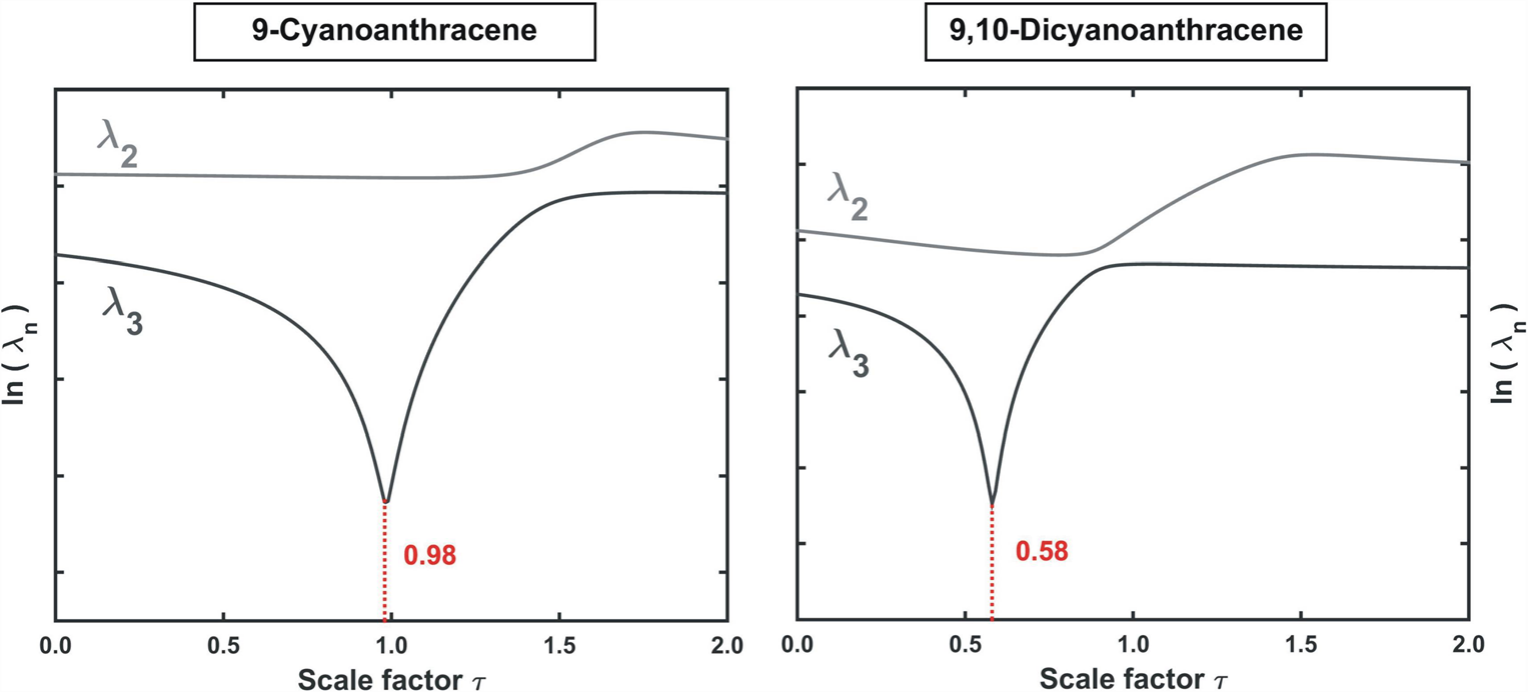
Graphical visualisation of the iterative RAFA algorithm. The singular values λ obtained for the difference matrices **D**_**MIX**_ **(12)** are plotted against a set of the corresponding scaling parameters τ used for their construction. The optimal value τ_0_ corresponds to a minimum value of the ‘last’ significant singular value (third). Just for comparison, the evolution of the second one is also shown. The applied logarithmic scale allows for easier observation of the extremes

With the use of the reference ‘idealised’ **Y**_**CNA**_ and **Y**_**DCNA**_ excitation-emission maps, the contribution to the recorded mixture signal of both CNA and DCNA **(12)** can be determined. The iterative RAFA algorithm (Fig. 12) shall be applied to find in the set of *τ* values the optimal scaling factor *τ*_*0*_, related to the minimum of the third (*f* = 3) singular value of the difference matrices

$$ {\mathbf{D}}_{\mathbf{MIX}}={\mathbf{X}}_{\mathbf{MIX}}-\tau \cdotp {\mathbf{Y}}_{\mathbf{CNA}}\kern1.33em \mathrm{and}\kern1.33em \mathbf{D}{\prime}_{\mathbf{MIX}}={\mathbf{X}}_{\mathbf{MIX}}-{\tau}^{\prime}\cdotp {\mathbf{Y}}_{\mathbf{D}\mathbf{CNA}} $$

In the result, two optimal scaling parameters of 0.98 and 0.58 are obtained for CNA and DCNA, respectively. Therefore, in order to determine the concentrations of these compounds in the analysed sample one needs to multiply τ_0_ values by the analyte concentrations *c*_*y*_ **(11)** in the calibration samples.

$$ {\displaystyle \begin{array}{c}{c}_x^{CNA}={\tau}_0\cdotp {c}_y^{CNA}=0.98\cdotp 0.50\ \left[{\mathrm{cm}}^3/10{\mathrm{cm}}^3\right]=0.49\ \left[{\mathrm{cm}}^3/10{\mathrm{cm}}^3\right]\\ {}{c}_x^{DCNA}={\tau}_0\cdotp {c}_y^{DCNA}=0.58\cdotp 0.50\ \left[{\mathrm{cm}}^3/10{\mathrm{cm}}^3\right]=0.29\ \left[{\mathrm{cm}}^3/10{\mathrm{cm}}^3\right]\end{array}} $$

Compared to the actual concentrations of CNA and DCNA in the mixture, equal to 0.50 and 0.30 [cm^3^/10 cm^3^] (see **SI - App. B 2.3**), respectively, the results are, to say the least, very satisfactory.

As it is demonstrated on the above example, the RAFA technique allows to independently determine the concentrations of the selected mixture constituents, without need of their physical separation. This is a great advantage in comparison to ‘traditional’ methods of quantitative analysis, as the separation of all mixture components is oftentimes difficult, time-consuming [2**–**3] and sometimes even impossible.

### In Search of the Signal Selectivity

In the case of the sample analysis when the number of preliminary information is strongly limited, a rather intuitive approach is to reduce the complex system to a set of one-component subsystems, for which the recorded signal would be selective. A search for such selective subsystems among the whole dataset can be conducted using certain techniques offered by factor analysis [18**–**20**,** 40].

As it was already proven, the number of significant singular values *λ* obtained for the data matrix **X**_**MIX**_ is strictly related to the total number of principal components attributed to the analysed system [5**,** 38]. The question which now should be addressed is whether or not there are any slices of the matrix, that are dominated by only one component. To find the answer, and ultimately to define the selective spectral regions of the EEM, the ‘whole’ matrix can be ‘sliced’ into smaller segments, for which a systematic analysis of the number of significant factors should be performed. As there are many hints suggesting how to systematically divide the ‘full’ data matrix into submatrices (i.e. [18**,** 40]), the Evolving Factor Analysis (EFA) [18**–**20] approach will be discussed here as an example.

**Fig. 13:**
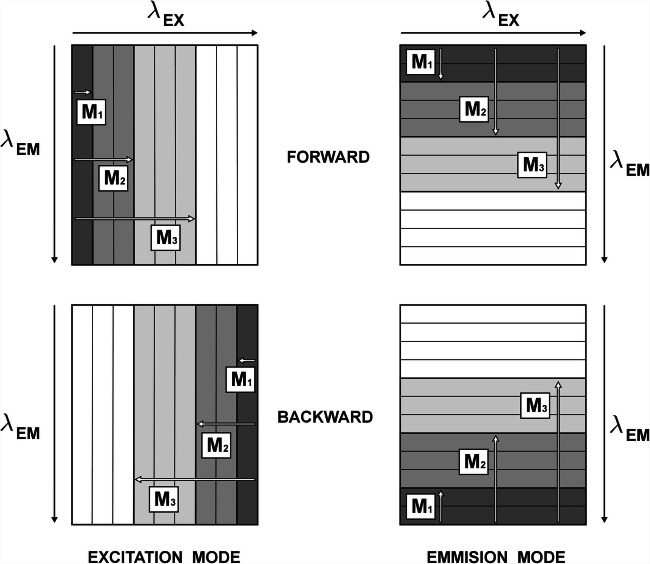
Schematic diagram of EFA. ‘Scanning’, that is stepwise augmentation of the analysed submatrix **M** may take place in two directions – forward or backward (from the shortest to longest wavelength or vice versa), and in two modes varying either the excitation or emission wavelength

Since the excitation-emission map can be viewed as a set of *n* fluorescence (or excitation) spectra, the initial submatrix **M**_**1**_ can be defined as its segment, consisting of ‘first’ *f* consecutive spectra, where *f* is the number of significant factors determined for the ‘whole’ original dataset **X**_**MIX**_. For this submatrix, the SVD procedure is performed, and *f* singular values *λ* are determined. On their basis it is possible to estimate how many significant factors are responsible for the variance of the currently analysed EEM segment. The second ‘slice’ **M**_**2**_ of the matrix **X**_**MIX**_ is then constructed by augmenting the submatrix **M**_**1**_ by one more consecutive spectrum (*f* + 1). Again, the SVD procedure is carried out. The cycle of augmenting the submatrix **M**_**i**_ (Fig. 12) and calculating its singular values *λ* is looped until the size of this expanding submatrix reaches the size of the original data matrix **X**_**MIX**_. The algorithm for systematic construction of submatrices may also be initiated from the ‘opposite side’ of the analysed data matrix. The matrix **M**_**1**_ would then consist of the ‘last’ *f* spectra (*n*, *n* - 1, …, *n - f* + 1) and it will be expanded to include the spectra localized on its ‘left’ side. To distinguish between this two equivalent ‘directions’ of the sumbatrix augmentation, the names ‘forward’ and ‘backward’ are used (Fig. 12) [19]. Moreover, the data matrix can be ‘sliced’ vertically as well as horizontally (Fig. 13). In the case of EEM it means that one of these modes would allow for the analysis of the spectral selectivity in the excitation while the other in the emission spectra.

**Fig. 14:**
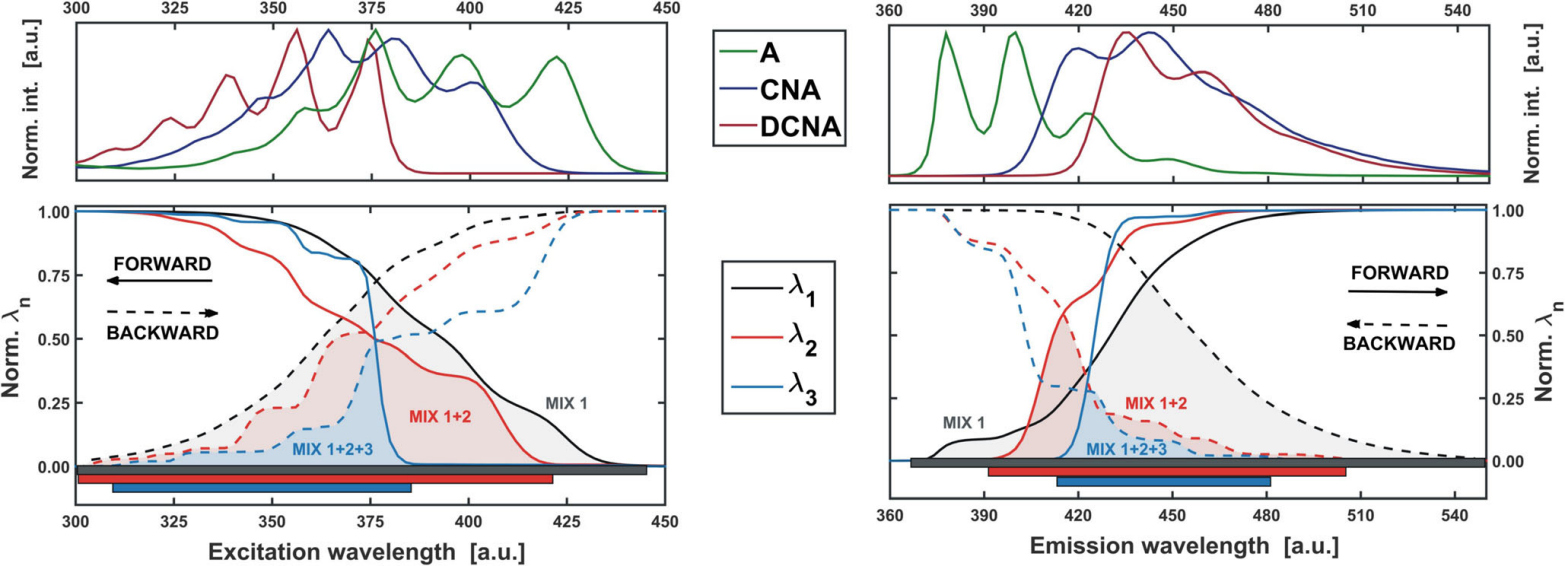
Curves normalized by their maximum values, showing the evolution of significant singular values of **X**_**MIX**_ as a function of the adopted range of the excitation (left) and emission wavelengths (right). Single component area is marked in grey (MIX 1), two component in red (MIX 1 + 2) and three component in blue (MIX 1 + 2 + 3). For comparison, at the top of the plots the spectra of individual mixture components are presented

Finally, by comparing significance of the singular values *λ*, obtained in each iteration, for instance graphically (see Fig. 14), the analysis of how the number of significant factors evolves with the size of the expanding submatrix (and thus with the wavelength range) can be done.

The example of the EFA procedure (see EFA routine - **SI**, App. **C.5**) will be illustrated here on the data matrix **X**_**MIX**_ (Fig. 8). The ‘scanning’ procedure was performed by augmenting an initial set of three (*f* = 3) emission (columns) and excitation (rows) spectra in both ‘forward’ and ‘backward’ directions (from ‘red-to-violet’ and from ‘violet-to red’, Fig. 13). The outcomes presenting the evolution of *λ* values in both excitation and emission wavelengths are displayed in Fig. 14. The interpretation of the presented plots is as follows. ‘Going forward’ from longer to shorter excitation wavelengths (‘red-to-violet’) it can be observed that up to 425 nm, only one *λ* is noticeably different from zero. Thus, the signal is selective in this range (DCNA). Then, the second singular value becomes significant (two components up to 385 nm), and finally, at the 385 nm wavelength – also the third. In the ‘backward’ direction, practically from the very beginning (300–305 nm) all three *λ* evolve simultaneously, which means that there is no selective range at the ‘violet edge’ of the mixture excitation spectrum.

Interpretation of the EFA plot for the emission spectra *is just analoguous*. However, in contrast to the excitation spectra, the backward EFA indicates that at the ‘very end’, the signal comes from only one component, which does not fully correspond to the reality (Fig. 7). A two-component signal, related to CNA and DCNA should be observed in the range of 505–550 nm (Fig. 14 – top panel). Unfortunately, the spectra of these two substances in this range remain practically identical and therefore, mathematically, the dataset is associated with only one component. This is a perfect example of one of the main problems encountered in factor analysis. Combining mathematical and chemical methods do not always has to be consistent.

**Fig. 15:**
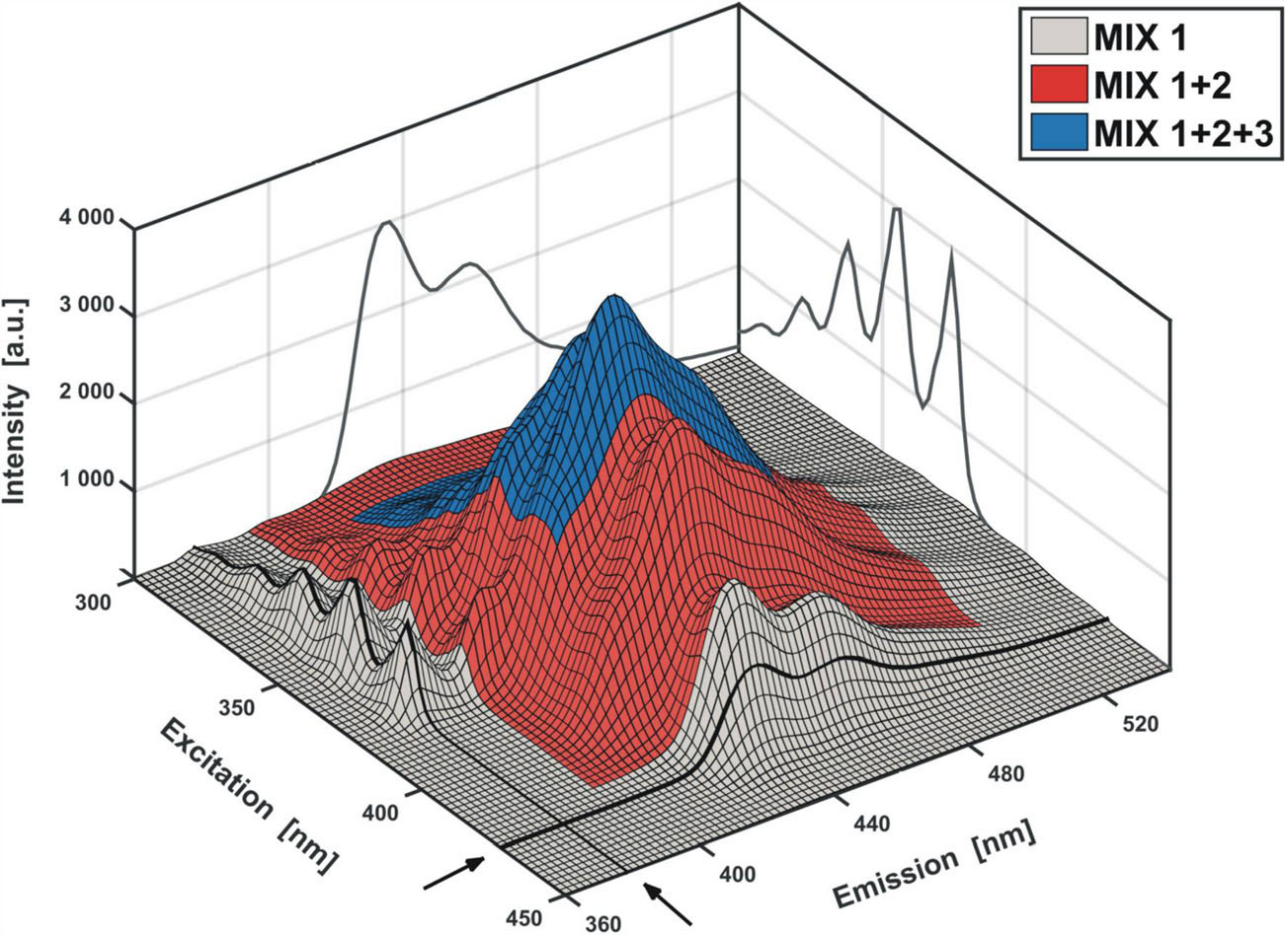
Excitation-emission map of the model mixture from Fig. 8 after determining the amount of significant factors by EFA method (Fig. 14). Spectra (black lines) coming from the marked in grey selective regions (MIX 1) are plotted on the side walls. Two-component regions are indicated in red (MIX 1 + 2) and three-component in blue (MIX 1 + 2 + 3)

Nevertheless, by combining the obtained results (colored surfaces in Fig. 14), the discussed EFA algorithm allows to determine in which regions of the excitation-emission map the recorded signal remains selective and how complex the other segments of EEM are (Fig. 15). As a result, the single-component spectral ranges may be picked out, which substantially facilitates the analysis of the studied system. In such a case the spectra of ‘pure’ components can directly be gathered into one block.

### Sample as a ‘Black Box’

When faced with ‘fully’ unknown samples, any technique allowing at least to estimate the individual excitation or emission spectra of its components is extremely useful. One such means is the Generalised Rank Anihilation Method (GRAM) [24**,** 25]. Because GRAM is an ‘extended’ version of the RAFA approach (**Chapter 4.3**) [23], the algorithm is in an analogous manner focused on finding such a transformation of the pair of the data matrices **X**_**MIX**_ and **Y**_**MIX**_, that would result in annihilation of the signal coming from one of the fluorescent species **(12)**.

Since GRAM, unlike ‘classical’ RAFA, enables determination of more than one component at the same time, the successful usage of this method calls for meeting another condition. Relative contributions of all components to the recorded signal have to be mutually different between the two compared samples. This condition is obeyed when the ratios of all the concentrations are different for **X**_**MIX**_ and **Y**_**MIX**_. However the required variability may be also fulfilled by addition of a small portion of a quencher to the examined mixture (see SI – Appendix B.4.3). According to the Stern-Volmer eq. **(****2****)**, the intensity of the emitted light will decrease for each fluorophor in a slightly different way (Fig. 8). Thus, the individual contributions of all components to the total spectrum would differ before and after the addition of the quencher.

For the considered example of a three-component model mixture, in terms of the individual signal annihilation, a set of three optimal scaling factors τ_0_ should be obtained **(8)**.

$$ {\mathbf{D}}_{\mathbf{MIX}}^{\mathbf{0}}={\mathbf{X}}_{\mathbf{MIX}}-{\tau}_0\cdotp {\mathbf{Y}}_{\mathbf{CNA}}={\mathbf{X}}_{\mathbf{A}}+{\mathbf{X}}_{\mathbf{D}\mathbf{CNA}} $$

**Fig. 16:**
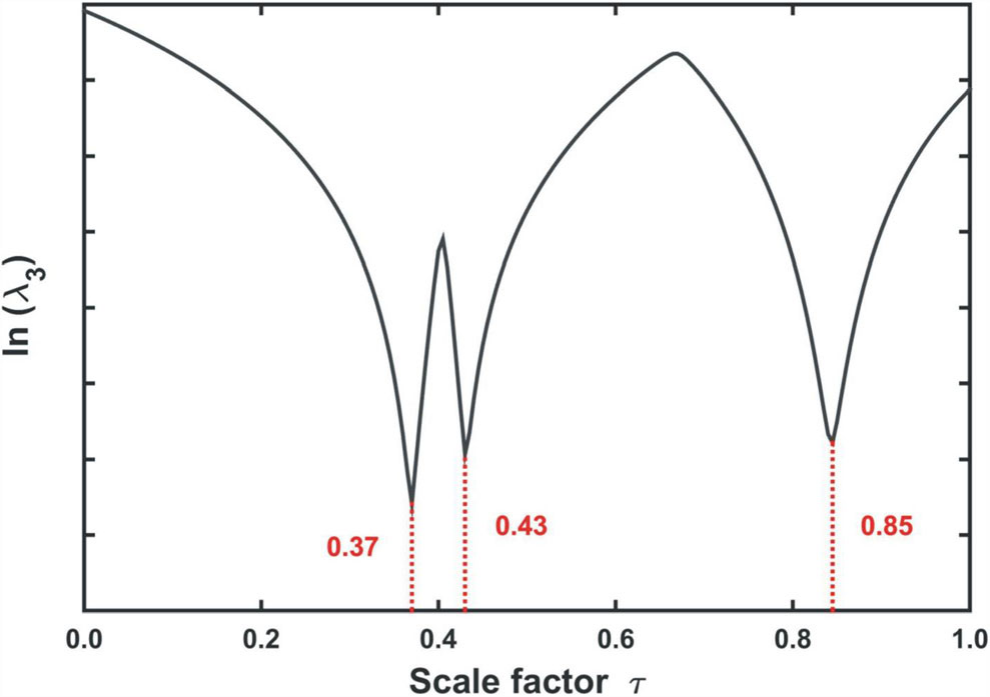
Graphical visualization of the iterative RAFA algorithm for a three-component system (A, CNA, and DCNA). The plot shows three minima of the third singular value. However, a direct correspondence with particular components is not established

These can be estimated by the iterative algorithm (Figs. 16, 12), already discussed in **Chapter 4.3** (RAFA), applied to a pair of the excitation-emission maps recorded before (**Y**_**MIX**_) and after (**X**_**MIX**_) the addition of KI (Fig. 8).

Although it can be clearly seen that the contributions of all three components (A, CNA, DCNA) to the variance of the resulting difference spectral matrix are successively ‘eliminated’, it is not possible to assign which *τ*_*0*_ value refers to which analyte. The obtained information seems to be rather ‘useless’ for the purpose of the quantitative analysis of the sample.

However, with the use of a ‘smart’ mathematical transformation of matrices **X**_**MIX**_ and **Y**_**MIX**_ it is possible to obtain the optimal scaling parameters *τ*_*0*_ and relate them to the excitation **S**_**EX**_ and emission **S**_**EM**_ spectra of all the components. This approach, known as non-iterative version of GRAM [23**–**25], consists of three main steps (**SI - App. A.5**). First, one of the data matrices (preferably the ‘reference’ one) is decomposed with the SVD algorithm (here it is **Y**_**MIX**_).

$$ {\mathbf{Y}}_{\mathbf{MIX}}=\mathbf{U}\boldsymbol{\Lambda } {\mathbf{V}}^{\mathbf{T}} $$

Next, from the second data matrix (here **X**_**MIX**_) and the truncated (*f* = 3) SVD matrices(Fig. 3), a helping square matrix **H** is formed **[**24**]**

$$ \mathbf{H}={\overline {\mathbf{U}}}^{\mathbf{T}}{\mathbf{X}}_{\mathbf{MIX}}\overline {\mathbf{V}}{\overline {\boldsymbol{\Lambda}}}^{-\mathbf{1}} $$

for which the eigenvector-eigenvalue problem is finally solved.

$$ \mathbf{Hr}={\tau}_0\mathbf{r} $$

The sequentially calculated eigenvalues are identical to the optimal scaling factors *τ*_*0*_ **(11)** (Fig*.* 16), while the set **R** of the associated eigenvectors **r** may be used to obtain the excitation and emission spectra of ‘pure’ components (**7c**, see **SI - App. A.5,** **[**24**]**).

$$ {\mathbf{S}}_{\mathbf{EM}}=\overline {\mathbf{U}}\mathbf{R} $$

$$ {\mathbf{S}}_{\mathbf{EX}}^{\mathbf{T}}={\mathbf{R}}^{-\mathbf{1}}\overline {\boldsymbol{\Lambda}}{\overline {\mathbf{V}}}^{\mathbf{T}} $$

The assignment of all *τ*_*0*_ values to all mixture components is then possible.

**Fig. 17:**
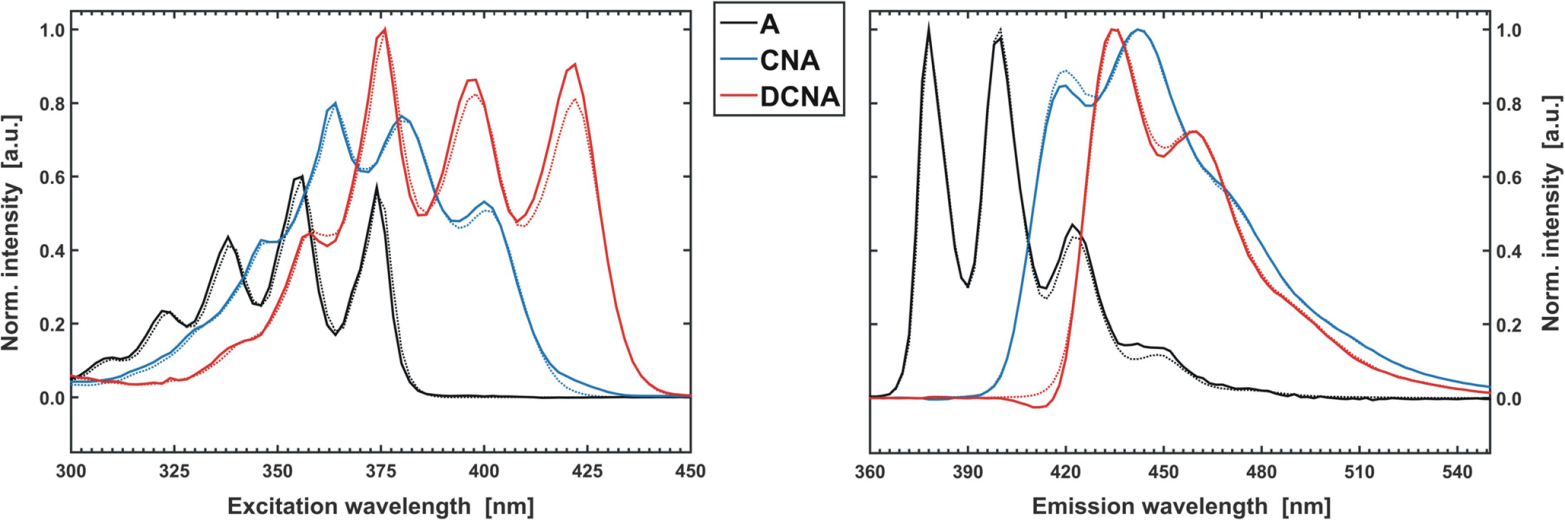
The fluorescence excitation (left) and emission spectra (right) obtained by GRAM technique (continuous lines) applied on the excitation-emission maps of the model mixture (Fig. 8). For the sake of comparison, the spectra measured individually for A, CNA and DCNA (dotted lines, Fig. 7) are also presented

The fluorescence emission and excitation spectra, ‘extracted’ from the model excitation-emission maps by the direct GRAM approach (see GRAM routine - **SI**, App. **C.6**), are presented in Fig. 17. As can be noticed, the calculated spectra exhibit a very high similarity to the spectra recorded for individual components.

This indicates that GRAM may be successfully used for both qualitative and quantitative analysis of complex mixtures. It is worth to note that for the former purpose no special conditions have to be fulfilled. In the latter case, however, a proper calibration sample has to be prepared (just like in the ‘classical’ RAFA), because the presented quencher addition technique is not suitable for determination of the absolute concentrations.

Eventually, it can be mentioned, that if needed, the spectra estimated by GRAM may be refined with some dedicated algorithms, allowing for example to remove (residual) negativities (i.e. ALS [41] – basics of the approach - see **SI -** App. **A.6,** routine – App. **C.7**).

### Factor Analysis in Physico-Chemical Studies

Since the methods of factor analysis are widely used in physicochemical studies of multi-component systems (i.e. in kinetics and thermodynamics) [42**–**45], at the very end of this article, an example of such application will be briefly discussed.

As far as the model system of three fluorophores (A, CNA, and DCNA) is concerned, the physicochemical characteristics may involve, for instance, an estimation of the Stern-Volmer quenching constants *K*_*SV*_ for each substance **(2)**. For that purpose, the decay of the individual emission intensity, caused by the addition of a quencher, should be evaluated. Determining the ratio of the fluorescence intensities measured before (*I*^0^_em_) and after (*I*^Q^_em_) the addition of a certain amount *Q* of the quencher **(2)**, brings into the scene the already discussed GRAM or RAFA approach (**Chapters 4.3** and **4.5**). To reduce the time consumption of the research, the spectral measurements can be made at only a few (here at least three, *f* = 3) excitation (or emission) wavelengths producing the spectra with a contribution from all three components (MIX 3 range in **Figs.** **14** and **15**). The excitation lines of 345, 355 and 365 nm may serve as an example (see SI – Appendix B.4.3). The fluorescence spectra are then measured for the unquenched sample and each time when a successive portion of the quencher *Q* is added to the mixture. As the result, the ‘reference’ matrix **Y**_**0**_ (*Q* = 0) as well as a set of consecutive **X**_**Q1**_, **X**_**Q2**_, **X**_**Q3**_ etc. data matrices are obtained. Using either iterative or direct version of GRAM, a set of optimal scaling parameters *τ*_*0*_ is determined for all the pairs of matrices **Y**_**0**_ and **X**_**Q**_ (X_Q_ = X_Q1_, X_Q2_, …).

$$ {\mathbf{D}}_{\mathbf{Q}}={\mathbf{X}}_{\mathbf{Q}}-\tau \cdotp {\mathbf{Y}}_{\mathbf{0}} $$

Due to the fact that **Y**_**0**_ is treated as the ‘reference’ matrix, the obtained parameters *τ*_*0*_ describe the ratio of the quenched (*I*^*Q*^_*em*_) to unquenched (*I*^*0*^_*em*_) fluorescence intensity for all the components at a certain level Q of the quencher concentration. Thus, the reciprocal values of *τ*_*0*_ are identical to the intensity ratios as defined by the Stern-Volmer eq. **(****2****)**.

$$ \frac{I_{em}^0}{I_{em}^Q}={\tau_0}^{-1}=1+{K}_{SV}\cdotp Q $$

**Fig. 18:**
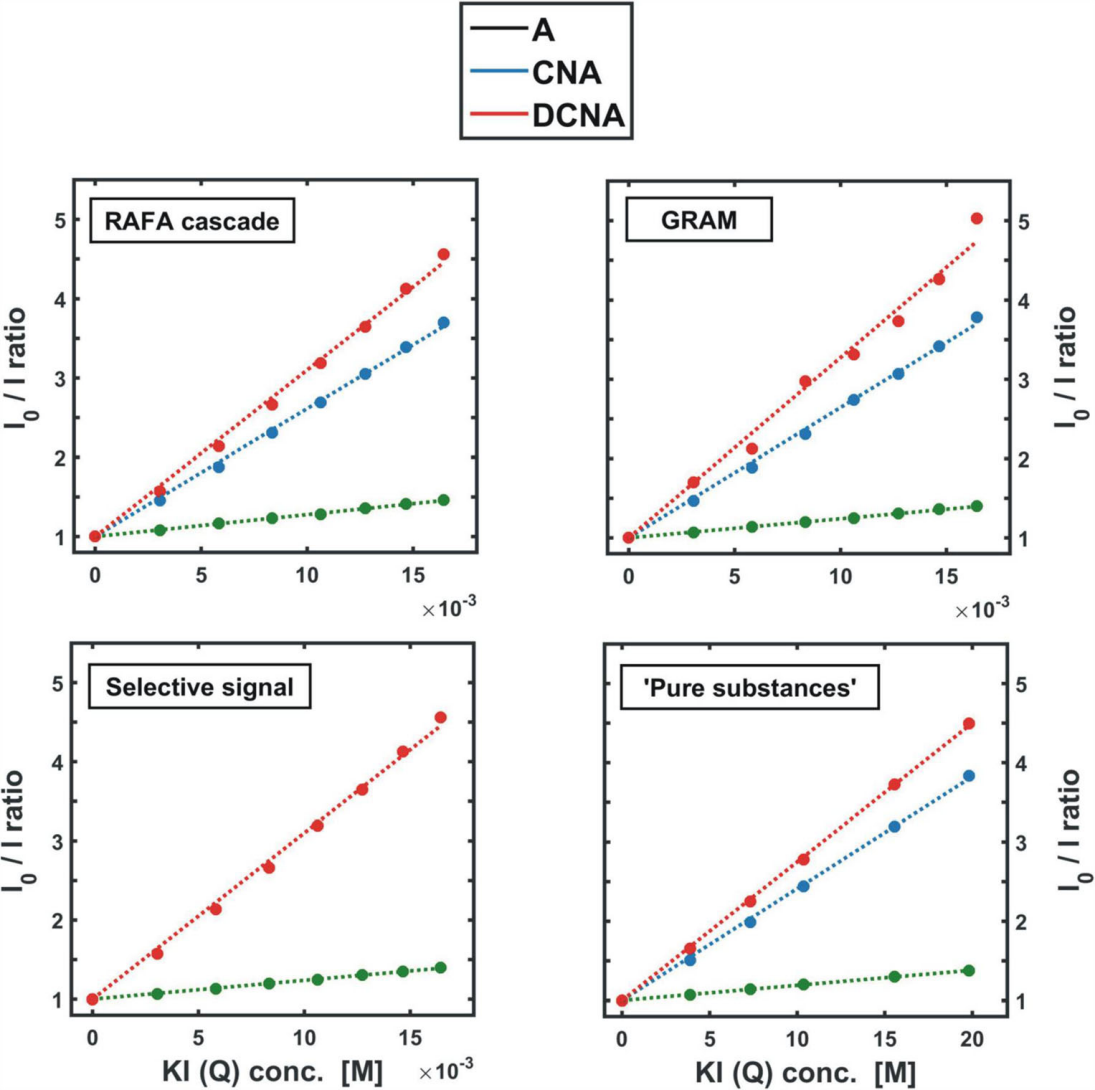
Stern-Volmer plots describing the fluorescence quenching process occurring in the analyzed mixture (Table 2). The data points were obtained by GRAM, RAFA ‘cascade’ and selective signal analysis. For comparison, the results of measurements conducted individually for ‘pure’ substances are also shown

Consequently, in order to determine the values of the Stern-Volmer quenching constants *K*_*SV*_, the reciprocals of *τ*_*0*_ are plotted against the quencher concentration *Q* (Fig. 18), and then a linear regression **(****2****)** is performed with a unit intercept. The slope of a straight line of best fit drawn through the data points determines the value of *K*_*SV*_ (Table 2). The full routine can be found in **SI**, as Appendix **C.8**.

**Table 2:** Stern-Volmer quenching constants K_SV_ **(2)** determined with use of GRAM, selective signal analysis, and ‘sequential RAFA ‘cascase’ techniques (Fig. 19). For comparison, the Stern-Volmer constants determined independently for each of the ‘pure’ components are also presented

–	GRAM	Selective signal	RAFA ‘cascade’	Pure substances
K_SV_ A [M^−1^]	24.12 ± 0.18	23.77 ± 0.19	27.74 ± 0.23	19.06 ± 0.04
K_SV_ CNA [M^−1^]	164.3 ± 1.7	- ^#2^	161.3 ± 1.3	141.2 ± 1.2
K_SV_ DCNA [M^−1^]	227.4 ± 5.4	210.1 ± 2.4	210.1 ± 2.4	175.0 ± 1.0
R^2^_min_^#1^	0.99766	0.99945	0.99945	0.99980

#1 - R^2^_min_ - the smallest value of the coefficient of determination R^2^ obtained for a given set of A, CNA and DCNA Stern-Volmer plots;

#2 – there is no selective region for CNA;

An alternative, though less ‘direct’ approach, is to obtain three sets of the fluorescence quenching spectra of single fluorophores. With the use of EFA (**Figs.** **14** and **15**) it can be noticed that both anthracene and 9,10-dicyanoanthracene exhibit selective emission in certain wavelength regions of the EEM. Therefore, by performing measurements under such spectral conditions, one can directly obtain a ‘pure’ signal of the quenched fluorescence for both A and DCNA (Fig. 18, Table 2). In this way, however, a selective signal for cyanoanthracene cannot be extracted. Thus, a more sophisticated method should be applied.

The EFA performed on the excitation-emission map reveals that the signal coming from CNA can be observed in some two-component regions (MIX 2, Fig. 15). As the spectra of fluorescence quenching of both A and DCNA are known, the RAFA (or GRAM) technique can now be used to eliminate the signal contribution from the counterpart fluorophore by its annihilation. Analogically, the same procedure can be applied to decompose spectra, where the signal comes from three components. An exemplary algorithm, allowing to obtain the series of the quenched fluorescence spectra of A, CNA and DCNA is presented below.

**Fig. 19:**
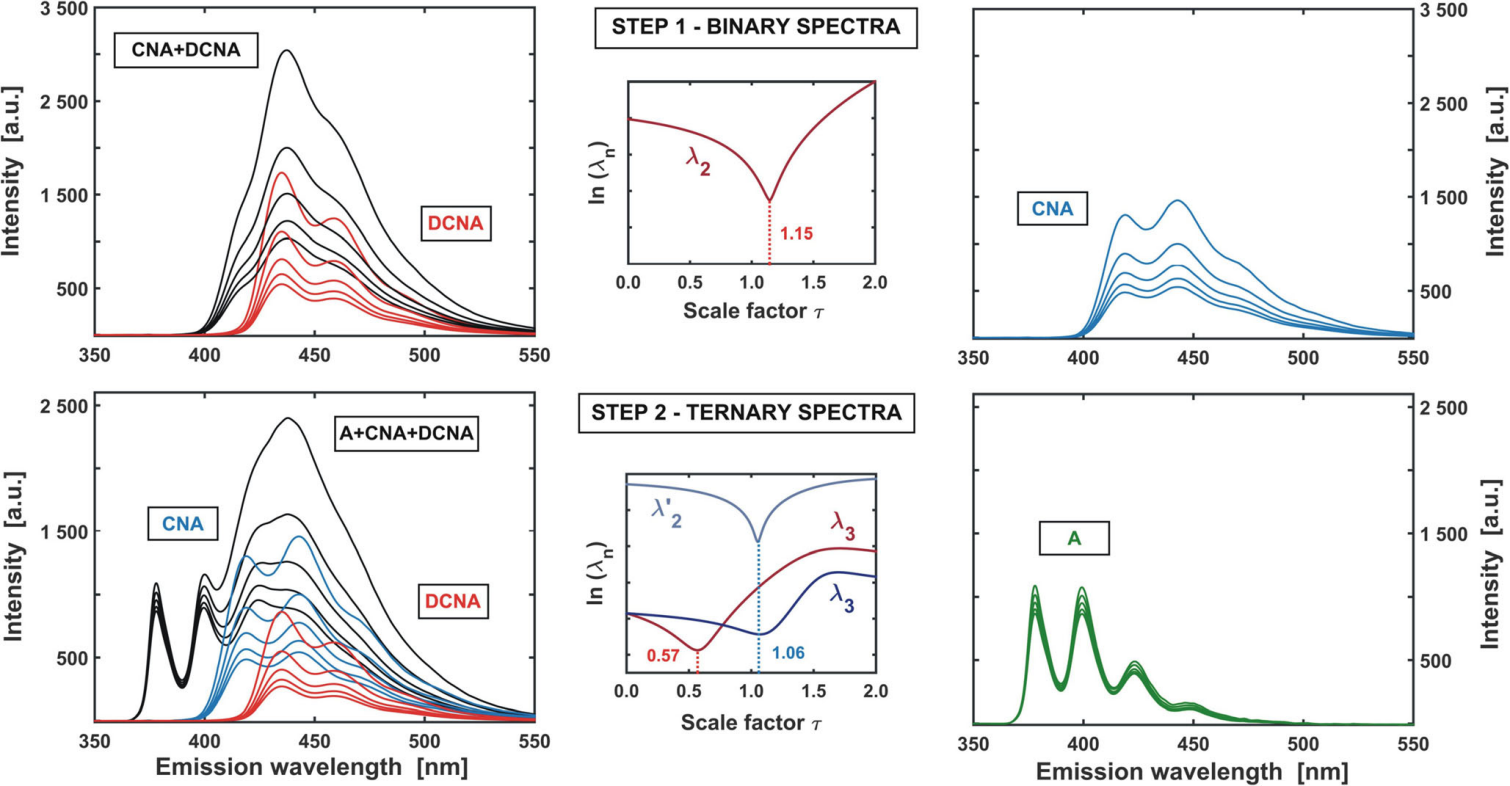
Example of ‘cascade’ RAFA procedure allowing to extract ‘pure’ spectra of all components by comparing three different datasets. In step one (upper panel), DCNA signal (recorded selectively) is annihilated (minimum of λ_2_) from a two-component spectral dataset. Thus, individual spectra of CNA are obtained. In step two, DCNA and CNA contributions are subtracted from ternary spectra. As a result, ‘pure’ spectra of A are recovered. The subtraction can be performed either sequentially or simultaneously. In the first case, a three-component spectral dataset is deprived of DCNA contribution - λ_3_ - and the resulting two-component spectral data matrix - of remaining CNA variance - λ_2_’. In the second case, the contributions of both DCNA and CNA to a ternary mixture spectral dataset are estimated directly - 2 x λ_3_)

1. The spectra are recorded in the region selective for DCNA, using excitation line 425 nm.
2. Simultaneously, the two-component fluorescence spectra (CNA + DCNA) are measured with the excitation wavelength of 400 nm and the three-component spectra (A + CNA + DCNA) as excited with the 355 nm line.
3. RAFA algorithm is applied on the first two datasets (minimum of the second singular value is searched). The resulting two-component spectra are then ‘purified’ from the signal contribution of DCNA. Consequently, the CNA spectra of quenched fluorescence are obtained (Fig. 19**–** step 1).
4. The annihilation of the DCNA signal contribution is then performed in an analogous manner for the three-component dataset (the evolution of the third singular value is traced). Then RAFA is repeated for the resulting two-component mixture (A + CNA, second singular value). As the CNA contribution disappears, the obtained spectra represent the ‘pure’ signal of A (Fig. 19 – step 2).

**4′.** Alternatively, one can simultaneously determine the spectral contribution of CNA and DCNA to the third spectral dataset (for both constituents the third singular value is traced independently). Then, both matrices, containing the ‘pure’ spectra of these compounds are subtracted from the data matrix containing the spectra of the three-component system. The final result should be identical as that in the previous step-wise approach.

When the above algorithm is completed (see routine App. **C.9** in **SI**), the three series of quenched fluorescence spectra of individual components are recovered from the multi-component dataset (Fig. 19). The Stern-Volmer plots are then obtained in the ‘classical’ way, that is by direct calculation of the proper ratios of the unquenched to quenched emission intensities (Fig. 18). The resulting Stern-Volmer constants *K*_*SV*_ can be found in Table 2.

Comparing the values of the Stern-Volmer constants obtained by GRAM, ‘cascade’ RAFA and selective region analysis (Table 2), it can be concluded that all these approaches remain consistent, as they provide very similar results.

What is worth to be noticed is the fact that the Stern-Volmer quenching constants estimated for all three components in a mixture are slightly different from those determined independently for single-component solutions (see **SI** – Appendix **B.4.2**). The higher values obtained in the case of the former ones may likely suggest, that some subtle, additional interactions between the molecules in the mixture occur. This effect usually evades observation when the system is separated into components in order to perform the analysis in a ‘traditional’ way.

The above example clearly shows that some phenomena unveiled by the methods of factor analysis remain ‘unavailable’ for classical analytical techniques.

## A Brief Summary

The main purpose of the examples discussed in this article was to highlight the opportunities and benefits of applying the chemometric methods in the everyday laboratory routine.

On a few practical examples it was shown that factor analysis techniques can be successfully used in order to a) estimate the number of components in the examined sample (PCA), b) search for the selective signal in the spectra of a mixture (EFA), c) validate whether the particular substance is present (or not) in the sample (TFA) and d) perform qualitative and quantitative analysis of the sample (RAFA & GRAM). It is worth to mention that all the results were obtained only by the computer analysis of the datasets, measured for the mixtures. No physical separation of the components was required at any step of the undertaken analysis, which gives an alternative to ‘traditional’ approaches such as chromatography and electrophoresis.

Although the potential offered by the recalled techniques is believed to be already noticed, it should be admitted that it is just a ‘tip of the iceberg’. Nowadays, the number of all available algorithms and their variants is practically countless. Moreover, the techniques may be combined together in both highly specific as well as general way, which only multiplies the total number of tools suitable for the analysis of spectral datasets offered by chemometrics.

Unfortunately, this ‘mathematized’ treatment of quantitative aspects of the spectroscopic data seems to be not so popular and sometimes even unknown within numerous communities of chemists and spectroscopists. Therefore, by publishing this article, the Authors hope to bring the factor analysis algorithms closer to creative individual researchers working in various domains of chemistry.

## Supplementary Information

ESM 1 (PDF 879 KB)

ESM 2 (ZIP 654 KB)

## Data Availability

All data generated or analysed during this study are included in supplementary information files (Appendix D) for this article.
