# Supplementary material for: Do Spectra Live in the Matrix? A Brief Tutorial on Applications of Factor Analysis to Resolving Spectral Datasets of Mixtures
Source: J Fluoresc. 2021 Aug 6;31(6):1599–616. doi: 10.1007/s10895-021-02753-w (PMC8547214; doi:10.1007/s10895-021-02753-w)
Supplement: Supplementary file 1 — (PDF 879 KB) [file 10895_2021_2753_MOESM1_ESM.pdf]

Journal of Fluorescence

Do spectra live in the matrix? A brief tutorial on applications of factor analysis to resolving spectral datasets of mixtures

Andrzej J. Kałka[1], Andrzej M. Turek*[2]

Faculty of Chemistry
Jagiellonian University in Kraków
2 Gronostajowa st.
30-387 Kraków
POLAND

1) andrzej.kalka@doctoral.uj.edu.pl

* 2) turek@chemia.uj.edu.pl

SUPPLEMENTARY INFORMATION (SI)

Appendix A..**pp. 2 – 9**

Selected mathematical and algorithmic issues

Appendix B..**pp. 10 – 13**

Experimental details

Appendix C..**pp. 14 – 21**

MATLAB codes

Appendix D.. **Appendix_D.zip**

Measured spectral datasets

APPENDIX A

Selected mathematical and algorithmic issues

1. Pseudo-inverse operations on rectangular matrices

The data matrix **X** can be written as a product of two rectangular matrices **S** and **C**:

$$\mathbf{X} = \mathbf{S} \cdot \mathbf{C}^{\mathrm{T}} \qquad \textit{(S. 1)}$$

where **T** denotes matrix transposition of the latter one. If all the matrices were square, in general it would be possible with use of well-defined algorithms to determine the inverse matrices $\mathbf{S}^{-1}$, meeting the condition:

$$\mathbf{S}\mathbf{S}^{-1} = \mathbf{1}$$

where **1** is an identity matrix. In such a case, **C** matrix would be determined with the following step.

$$\mathbf{S}^{-1}\mathbf{X} = \mathbf{S}^{-1}\mathbf{S}\mathbf{C}^{\mathrm{T}} = \mathbf{1} \cdot \mathbf{C}^{\mathrm{T}} = \mathbf{C}^{\mathrm{T}}$$

For rectangular matrices, however, the concept of the inverse matrix is not valid. Therefore, a certain 'mathematical trick' allows to determine a matrix that fulfills the analogous requirements. Using the fact that the product of any matrix and its transposition ($\mathbf{S}\mathbf{S}^{\mathrm{T}}$ or $\mathbf{S}^{\mathrm{T}}\mathbf{S}$) is always a square matrix, the equation under consideration **(S. 1)**

$$\mathbf{X} = \mathbf{S} \cdot \mathbf{C}^{\mathrm{T}}$$

may be multiplied by an appropriate combination of matrix **S** and its transposition.

$$\{(\mathbf{S}^{\mathrm{T}}\mathbf{S})^{-1}\mathbf{S}^{\mathrm{T}}\}\mathbf{X} = \{(\mathbf{S}^{\mathrm{T}}\mathbf{S})^{-1}\mathbf{S}^{\mathrm{T}}\}\mathbf{S} \cdot \mathbf{C}^{\mathrm{T}} = (\mathbf{S}^{\mathrm{T}}\mathbf{S})^{-1}(\mathbf{S}^{\mathrm{T}}\mathbf{S})\mathbf{C}^{\mathrm{T}} = \mathbf{1} \cdot \mathbf{C}^{\mathrm{T}} = \mathbf{C}^{\mathrm{T}}$$

As the result, a 'recipe' for calculating the $\mathbf{C}^{\mathrm{T}}$ matrix is obtained. The used above product

$$(\mathbf{S}^{\mathrm{T}}\mathbf{S})^{-1}\mathbf{S}^{\mathrm{T}} = \mathbf{S}^{+} \qquad \textit{(S. 2)}$$

for which the following condition is hold

$$\mathbf{S}\mathbf{S}^{+} = \mathbf{1}$$

is called the (left) pseudo-inverse of the matrix **S**.

Exactly the same procedure can be performed for $\mathbf{C}^{\mathrm{T}}$ matrix. One gets then the definition

$$\mathbf{C}^{\mathrm{T}}(\mathbf{C}\mathbf{C}^{\mathrm{T}})^{-1} = \mathbf{C}^{+}$$

of the (right) pseudo-inversion of matrix $\mathbf{C}^{\mathrm{T}}$, that can be used to recover matrix **S** from the equation **(S 1)**.

2. Properties of the Singular Value Decomposition

Practically all data matrices **X** with dimensions *m* by *n* ($m \geqslant n$, *m* rows and *n* columns) can be decomposed into a product of three matrices **U**, **Λ** and **V**.

$$\mathbf{X} = \mathbf{U\Lambda V^T}$$

Figuratively, this procedure resembles solving the 'generalised' eigenvector-eigenvalue problem for a square matrix **A** (case *m* = *n*)

$$\mathbf{Ag_i} = \gamma_i \mathbf{g_i}$$

consisting in finding the set **Γ** of all eigenvalues γ_i together with associated to them eigenvectors $\mathbf{g_i}$ meeting the above condition.

Unfortunately, if the matrix **X** is not square (*m* > *n*), the 'classical' eigenvector-eigenvalue problem remains ill-defined. However, the rectangular matrix **X** can somehow be 'transformed' to a square form by performing the following multiplication:

$$\mathbf{X^T X} \quad \text{lub} \quad \mathbf{XX^T}$$

These products, with the dimensions *n* x *n* ($\mathbf{X^T X}$) and *m* x *m* ($\mathbf{XX^T}$), are called the small and large covariance matrix, respectively.

The solution of the eigenvector-eigenvalue problem for both covariance matrices will be, respectively, two sets of **U** and **V** eigenvectors and a 'common' set of eigenvalues **Γ**.

$$\mathbf{X^T X V} = \mathbf{\Gamma V}$$

$$\mathbf{XX^T U} = \mathbf{\Gamma U}$$

Exactly the same set of eigenvectors **U** and **V** is obtained by using the SVD algorithm.

$$\mathbf{X} = \mathbf{U\Lambda V^T}$$

However, the **Λ** matrix does not contain 'directly' eigenvalues γ_i, but singular values λ_i instead. To see how these values are related, an important property of eigenvectors has to be mentioned. Both $\mathbf{u_i}$ and $\mathbf{v_i}$ vectors are characterized by orthonormality - apart from mutual orthogonality, they are additionally normalized to unit length. Their mutual scalar product will therefore take a unit or zero value.

$$\mathbf{v_i} \circ \mathbf{v_j} = \mathbf{v_i^T v_j} = 0, \qquad i \neq j$$
$$\mathbf{v_i} \circ \mathbf{v_j} = \mathbf{v_i^T v_j} = 1, \qquad i = j \qquad \textit{(S. 3)}$$

The above property, can be rewritten in matrix form as:

$$\mathbf{VV^T} = \mathbf{1} \quad \text{and} \quad \mathbf{UU^T} = \mathbf{1} \quad .$$

where **1** is the identity matrix. The SVD of the covariance matrix (small or large) will then be as follows

$$\mathbf{XX^T} = \mathbf{U\Lambda V^T}(\mathbf{U\Lambda V^T})^\mathbf{T} = \mathbf{U\Lambda V^T V\Lambda U^T} = \mathbf{U\Lambda}^2\mathbf{U^T}$$

After simple transformations on both sides of the above equation one gets

$$\mathbf{XX^T U} = \mathbf{U\Lambda}^2\mathbf{U^T U} = \mathbf{U\Lambda}^2 = \mathbf{\Lambda}^2\mathbf{U}$$

Comparing this expression with the eigenvector-eigenvalue problem of the $\mathbf{XX^T}$ covariance matrix

$$\mathbf{XX^T U} = \mathbf{\Gamma U}$$

the following dependency is derived

$$\mathbf{\Lambda}^2 = \mathbf{\Gamma} \qquad \textit{(S. 4)}$$

Consequently, the singular values λ_i obtained by SVD of the matrix **X** are equal to the square roots of the eigenvalues γ_i of the covariance matrix $\mathbf{XX^T}$ (or $\mathbf{X^T X}$ equivalently).

Finally, it is worth noting that the total number of singular values obtained by SVD is equal to the smaller dimension of the decomposed rectangular matrix. By convention, singular values are have nonnegative sign and the SVD algorithm usually sorts / ranks all λ_i in descending order (and thus also the associated eigenvectors).

3. Abstract and real spectra

Any $\mathbf{x_n}$ spectrum of a mixture of several components (A, B, C, ...), can be described as the appropriate sum of the spectra $\mathbf{s_A}$, $\mathbf{s_B}$, $\mathbf{s_C}$, ..., of 'pure' substances A, B, C, etc.

$$\mathbf{x_n} = a_n \cdot \mathbf{s_A} + b_n \cdot \mathbf{s_B} + c_n \cdot \mathbf{s_C} + ...$$

At the same time, each spectrum from the matrix **X** (column oriented) can be presented as a linear combination of **u**-type eigenvectors, provided by SVD.

$$\mathbf{x_n} = \alpha_n \cdot \mathbf{u_1} + \beta_n \cdot \mathbf{u_2} + \gamma_n \cdot \mathbf{u_3} + ...$$

Comparing the two expressions, it can be seen that $\mathbf{u_i}$'s eigenvectors (called abstract spectra), and real $\mathbf{s_i}$ spectra are each other reversible projections - the former can be obtained through a linear combination (sum and difference) of the latter. This dependence remains, of course, reflexive.

The determination of coefficients α, β and γ is quite simple thanks to the mutual orthonormality of eigenvectors **(S. 3)**. For example, in order to determine the value of α, a scalar product is calculated as follows:

$$\mathbf{x_n} \circ \mathbf{u_1} = \alpha_n \cdot \mathbf{u_1} \circ \mathbf{u_1} + \beta_n \cdot \mathbf{u_2} \circ \mathbf{u_1} + \gamma_n \cdot \mathbf{u_3} \circ \mathbf{u_1} + \ldots$$

or in an equivalent notation:

$$\mathbf{u_1^T x_n} = \alpha_n \cdot \mathbf{u_1^T u_1} + \beta_n \cdot \mathbf{u_1^T u_2} + \gamma_n \cdot \mathbf{u_1^T u_3} + \ldots$$

Taking into account the orthonormality of **u** vectors one gets:

$$\mathbf{u_1^T x_n} = \alpha_n \cdot 1 + \beta_n \cdot 0 + \gamma_n \cdot 0 + \ldots$$

and consequently

$$\alpha_m = \mathbf{u_1^T x_m} = \mathbf{u_1} \circ \mathbf{x_m}$$

Coefficients of linear combination of the abstract spectra to reproduce the individual spectra of the 'pure' components, $\mathbf{s_A}$, $\mathbf{s_B}$, $\mathbf{s_C}$, ... are calculated as 'special' case of the above approach ($\mathbf{x_n} = \mathbf{s_A}$).

As the real spectra are in general not orthogonal to each other, the determination of coefficients *a*, *b*, *c*, etc. has to be done differently. The least-squares regression methods are then most often used. Not involved in the details **[5]**, it can only be pointed out that the result is obtained by the following matrix transformation

$$[a_n, b_n, c_n \ldots] = \mathbf{S^+ x_n}$$

where **S** is the matrix containing a set of the 'pure' substances spectra. In the same way, 'recipe' of the abstract spectra can be found:

$$[a_i, b_i, c_i \ldots] = \mathbf{S^+ u_i}$$

An illustrative example of mutual dependency between real and abstract spectra will be provided for the data matrix from **Chapter 4.1**. Linear relationship between these two types of vectors **(Fig. 9)** are given by:

$$\mathbf{u_1} = 1.24 \cdot \mathbf{s_A} + 4.69 \cdot \mathbf{s_{CNA}} - 3.71 \cdot \mathbf{s_{DCNA}}$$
$$\mathbf{u_2} = 2.69 \cdot \mathbf{s_A} + 0.16 \cdot \mathbf{s_{CNA}} - 1.01 \cdot \mathbf{s_{DCNA}}$$
$$\mathbf{u_3} = 1.13 \cdot \mathbf{s_A} - 0.78 \cdot \mathbf{s_{CNA}} - 0.66 \cdot \mathbf{s_{DCNA}}$$

In can be clearly seen that the **u**-eigenvectors are strongly correlated with the real spectra (compare i.e. extrema in **Fig. 9** with bands maxima of real spectra), but are characterized by both negative and positive signs. So, the ethymology of the phrase 'abstract spectra' becomes now self-explanatory.

4. Exemplary statistical criteria used for principal factors determination

As it is shown in **Chapter 4.1**, estimation of the number of significant factors for the analysed dataset can be performed by simple comparison of the consecutive singular values, obtained by SVD. However, it may be helpful to make use of some statistically justified criteria. Among the huge variety of these **[42]**, the 'simplest' seems to be the relative variance σ^2_f of a given factor f (variable). Since the squares of the singular values λ are directly proportional to the variance of the data **(S. 4)**, the relative variance σ^2_f can be defined as a ratio of the f-th eigenvalue γ_f to the sum of all n obtained eigenvalues.

$$\sigma_f^2 = \frac{\gamma_f}{\sum_{i=1}^{n} \gamma_f} = \frac{\lambda_f^2}{\sum_{i=1}^{n} \lambda_i^2} \qquad \textbf{\textit{(S. 5)}}$$

Another criterion is the percentage of the total variance explained by the first f factors. Mathematically, it would be a sum of first f relative variances σ^2_i.

$$\Sigma_f = \frac{\sum_{i=1}^{f} \lambda_i^2}{\sum_{j=1}^{n} \lambda_j^2} = \sum_{i=1}^{f} \sigma_i^2 \qquad \textbf{\textit{(S. 6)}}$$

For more sophisticated and thus sometimes more powerful statistical criteria, the Reader is refereed to the proper literature, especially to the paper by Malinowski **[42]**.

5. Non-iterative algorithm of the rank annihilation factor analysis (GRAM)

The presented below derivation of the RAFA and GRAM non-iterative algorithms is based on the original work by Sanchez and Kowalski **[26]**.

Any data matrix, obtained from measurements of the excitation-emission maps for a single substance A, can be presented as the outer product of two vectors: the 'standard' $\mathbf{s}_{\mathbf{EM,\,A}}$ emission spectrum, and the transposed 'standard' $\mathbf{s}_{\mathbf{EX,\,A}}$ excitation (or absorption) spectrum, multiplied by the scaling factor c_A, having a sense of substance A concentration.

$$\mathbf{X}_{\mathbf{A}} = \mathbf{s}_{\mathbf{EM,\,A}}\, c_A\, \mathbf{s}^{\mathbf{T}}_{\mathbf{EX,\,A}}$$

In the case of a f -component mixture X (A, B, C ...), the $\mathbf{X_{MIX}}$ data matrix will be the sum of the respective single-component matrices:

$$\mathbf{X_{MIX}} = \mathbf{X_A} + \mathbf{X_B} + \mathbf{X_C} + ...$$

At the same time, it can also be presented as a product of three matrices:

$$\mathbf{X_{MIX}} = \mathbf{S_{EM} C_X S_{EX}^T}$$

The spectra of the individual components are then collected in the $\mathbf{S_{EX}}$ i $\mathbf{S_{EM}}$ matrices and the concentration scaling factors forms the diagonal, f x f sized, matrix $\mathbf{C_X}$.

If an another excitation-emission map is recorded, this time for mixture Y, the $\mathbf{Y_{EEM}}$ matrix shall be obtained. Let it be assumed, that Y sample is characterised by identical qualitative composition as X but concentrations of (all) the f individual substances remains different. Consequently, $\mathbf{Y_{MIX}}$ matrix can be presented as a combination of identical sets of $\mathbf{S_{EX}}$ and $\mathbf{S_{EM}}$ spectra and slightly modified diagonal $\mathbf{C_Y}$ matrix.

$$\mathbf{Y_{MIX}} = \mathbf{S_{EM} C_Y S_{EX}^T}$$

Upon transformation of both expressions to the form of

$$\mathbf{X_{MIX}} (\mathbf{S_{EX}^T})^+ \mathbf{C_Y} = \mathbf{S_{EM} C_X C_Y}$$

$$\mathbf{Y_{MIX}} (\mathbf{S_{EX}^T})^+ \mathbf{C_X} = \mathbf{S_{EM} C_Y C_X}$$

and comparison of both expressions, the following relationship is obtained

$$\mathbf{X_{MIX}} (\mathbf{S_{EX}^T})^+ \mathbf{C_Y} = \mathbf{S_{EM} C_X C_Y} = \mathbf{Y_{MIX}} (\mathbf{S_{EX}^T})^+ \mathbf{C_X}$$

Substituting $(\mathbf{S^T_{EX}})^+ = \mathbf{Z}$**,** $\mathbf{X_{MIX}} = \mathbf{X}$, and $\mathbf{Y_{MIX}} = \mathbf{Y}$, for the sake of clarity, results in

$$\mathbf{XZC_Y} = \mathbf{YZC_X}$$

SVD performed on one of matrices **X** or **Y** (here it is **X**), and truncrated to f significant factors:

$$\mathbf{X} \approx \bar{\mathbf{X}} = \bar{\mathbf{U}} \bar{\mathbf{\Lambda}} \bar{\mathbf{V}}^\mathbf{T}$$

gives

$$\bar{\mathbf{U}} \bar{\mathbf{\Lambda}} \bar{\mathbf{V}}^\mathbf{T} \mathbf{ZC_Y} = \mathbf{YZC_X}$$

Upon another substitution

$$\mathbf{Z*} = \bar{\mathbf{\Lambda}} \bar{\mathbf{V}}^\mathbf{T} \mathbf{Z}$$

$$\mathbf{Z} = \bar{\mathbf{V}} \bar{\mathbf{\Lambda}}^{\mathbf{-1}} \mathbf{Z*}$$

the above equation turns into

$$\bar{\mathbf{U}} \mathbf{Z*C_Y} = \mathbf{Y} \bar{\mathbf{V}} \bar{\mathbf{\Lambda}}^{\mathbf{-1}} \mathbf{Z*C_X}$$

Then, after multiplying both sides of the equation by $\mathbf{U}^{\mathbf{T}}$ and $\mathbf{C_X}^{-1}$

$$\mathbf{Z}^*\mathbf{C}_Y\mathbf{C}_X{}^{-1} = \bar{\mathbf{U}}^{\mathrm{T}}\mathbf{Y}\bar{\mathbf{V}}\bar{\mathbf{\Lambda}}^{-1}\mathbf{Z}^*$$

a classic form of the square matrix eigenvector-eigenvalue problem is obtained.

$$(\bar{\mathbf{U}}^{\mathrm{T}}\mathbf{Y}\bar{\mathbf{V}}\bar{\mathbf{\Lambda}}^{-1})\mathbf{Z}^* = (\mathbf{C}_Y\mathbf{C}_X{}^{-1})\mathbf{Z}^*$$

Defining the helping matrix **H** as

$$\mathbf{H} = \bar{\mathbf{U}}^{\mathrm{T}}\mathbf{Y}\bar{\mathbf{V}}\bar{\mathbf{\Lambda}}^{-1}$$

it can better illustrated by

$$\mathbf{H}\mathbf{z}_i^* = \tau_0^i\mathbf{z}_i^*$$

The resulting set of the eigenvalues ($\mathbf{C_Y C_X}^{-1}$) is identical to that known from iterative version of the algorithm providing the concentration ratios $\tau_0 = c_Y/c_X$ for each substance (A, B, C, ...) in both samples **(11)**.

In addition, a set **Z*** of the associated eigenvectors **z*** is also returned, through which the emission and excitation spectra of each substance can be determined. For this purpose, let the definitions of **Z** and **Z*** be reminded:

$$\mathbf{Z}^* = \bar{\mathbf{\Lambda}}\bar{\mathbf{V}}^{\mathrm{T}}\mathbf{Z} = \bar{\mathbf{\Lambda}}\bar{\mathbf{V}}^{\mathrm{T}}(\mathbf{S}_{\mathbf{EX}}^{\mathbf{T}})^+$$

The 'recipe' for calculating the excitation spectra will therefore be expressed as:

$$\mathbf{S}_{\mathbf{EX}}^{\mathbf{T}} = (\bar{\mathbf{V}}\bar{\mathbf{\Lambda}}^{-1}\mathbf{Z}^*)^+$$

Due to properties of (pseudo)inversion, this can be rewritten in the form of:

$$\mathbf{S}_{\mathbf{EX}}^{\mathbf{T}} = (\bar{\mathbf{V}}\bar{\mathbf{\Lambda}}^{-1}\mathbf{Z}^*)^+ = (\mathbf{Z}^*)^+(\bar{\mathbf{\Lambda}}^{-1})^+(\bar{\mathbf{V}})^+$$

Taking into account that **Z*** and $\mathbf{\Lambda}$ are square matrices and **V** is an orthogonal matrix ($\mathbf{V^T V} = \mathbf{1}$), their pseudoinvesions **(S.2)** are equivalent to ‘classic’ matrix inversions and transposition, respectively. This leads to the final expression given as:

$$\mathbf{S}_{\mathbf{EX}}^{\mathbf{T}} = (\mathbf{Z}^*)^{-1}\bar{\mathbf{\Lambda}}\bar{\mathbf{V}}^{\mathbf{T}}$$

The emission spectra can be obtained through a sequence of 'backward' substitutions:

$$\mathbf{S}_{\mathbf{EM}}\mathbf{C}_{\mathbf{X}} = \mathbf{X}(\mathbf{S}_{\mathbf{EX}}^{\mathbf{T}})^+ = \bar{\mathbf{U}}\bar{\mathbf{\Lambda}}\bar{\mathbf{V}}^{\mathrm{T}}(\bar{\mathbf{V}}\bar{\mathbf{\Lambda}}^{-1}\mathbf{Z}^*) = \bar{\mathbf{U}}\mathbf{Z}^*$$

Since all values of the diagonal matrix $\mathbf{C_X}$ are known, the emission spectra $\mathbf{S_{EM}}$ can be calculated as:

$$\mathbf{S_{EM}} = \bar{\mathbf{U}}\ \mathbf{Z}^*\ \mathbf{C}_x^{-1}$$

While deriving the above equations, it was assumed that both X and Y samples had the same composition in terms of quality. However, the algorithm is also suitable for cases where the two samples differ in the amount of ingredients. Generally, SVD is then carried out on the data matrix obtained for a mixture with more components. For details, however, the Reader is referred to the original article on 'direct' GRAM **[26, 27]** and RAFA **[25]** algorithms.

6. A very brief description of the ALS spectra refinement method

At the very end it can be mentioned, that in case of need, the spectra estimated by factor analysis methods be refined with some dedicated algorithms, allowing for example to remove (residual) negativities. One of them is Alternating Least Squares (ALS) approach **[45]**. Its principle of operation can be presented on example of EEM as alternating calculation of excitation and emission spectra, starting from the initial estimation, with looped cycle of formulas:

$$\mathbf{S}_{EX}^{T}{}' = \mathbf{S}_{EM}{}^{+}\mathbf{X}_{MIX}$$
$$\mathbf{S}_{EM}{}' = \mathbf{X}_{MIX}(\mathbf{S}_{EX}^{T})^{+}$$

In each iteration, resulting spectra are corrected for the applied constrains (i.e. for negativity by zeroing negative values).

$$\mathbf{S}_{EM} \geq 0; \quad \mathbf{S}_{EX}^{T} \geq 0$$

As the whole procedure is based on the least squares method, theoretically, with each cycle the calculated spectra gets closer to the optimum solution, that is spectra of the pure components.

APPENDIX B

Experimental details

1. Substances used in experiments

Measurements of the excitation and emission spectra were carried out for fluorophores which easy to obtain in affordable price such as anthracene (A; CAS: 120-12-7) and its derivatives: 9-cyanoanthracene (CNA, CAS: 1210-12-4), 9,10-dicyanoanthracene (DCNA, CAS: 1217-45-4) and 9,10-diphenylanthracene (DPhA, CAS: 1499-10-1).

Solutions of the above substances were prepared in methanol distilled prior to usage (MeOH, HPLC Grade). Potassium iodide (KI, CAS: 7681-11-0) was used as a quencher.

2. Preparation of solutions used in measurements

2.1 Stock solutions

In order to prepare the stock solutions, first the saturated solutions of all fluorophores in methanol were made. Except for anthracene, theese are characterised by maximal absorbance close to unity. The anthracene solution was therefore diluted to a similar absorbance value.

2.2 Single component solutions

Reference solutions of single components were obtained by diluting the stock solutions c.a. tenfold. Thus, their absorbance at level $A_{max} \approx 0.1$ allowed to maintain a linear fluorescence intensity to concentration ratio, according to Parker's law [35]. Following the principles of 'green chemistry', the total amount of consumed organic solvents was reduced to a minimum by using measuring flasks with as small capacity as possible (5 mL).

Standard calibration solutions of CNA and DCNA were also prepared by diluting exact 0.25 mL volume of the stock solutions to a total volume of 5 mL.

For the purpose of individual determination of the Stern-Volmer quenching constants, the 0.06 M methanol solution of potassium iodide was also prepared by dissolving a suitable portion of the solid salt in a small amount of methanol (about 0.100 g KI/ 10 mL MeOH).

2.3 Mixture of three fluorophores

The three-component mixture was prepared in such a way that its maximum absorbance did not exceeded a limit value of A equal 0.1 (Parker's law [35]). This was achieved by mixing the 0.4, 0.5 and 0.3 mL volumes of solutions of A, CNA and DCNA, respectively, (1.2 mL in total), and diluting the mixture to a total volume of 10 mL.

For the purpose of quenched fluorescence measurements, a 0.06 M solution of potassium iodide was made. In this case, however, the proper portion of KI (0.050 g) was dissolved not in the pure methanol, but in 5 mL of a freshly prepared three-component mixture.

3. Measuring instrumentation

Measurements of the absorption and emission spectra were carried out on a Hitachi dual-beam spectrophotometer U-2900 and a Hitachi F-7000 spectrofluorometer, respectively. Quartz cells with a 10 mm long optical path were used.

4 Spectral measurements

4.1 Absorption spectra

Measurements of the absorption spectra were made for stock solutions (unitary absorbance) and for the three-component mixture in the range of 300 - 460 nm. Pure methanol was used as a reference.

4.2 Measurements of emission spectra for pure substances

Fluorescence spectra were measured for solutions of all pure components obeying Parker's law ($A \approx 0.1$) in the range of 350 - 600 nm. On the basis of the previously recorded absorption spectra, the optimum excitation wavelength was selected for each substance. Excitation line was set at the maximum of the most intense vibrational band, sufficiently 'distant' from the red edge of the

absorption spectrum (so that the Rayleigh scattering band in the fluorescence spectra was not present). On the basis of the obtained fluorescence spectra, the appropriate emission wavelength was selected for each sample following analogous criteria, and the excitation spectra were measured in the same range as the absorption spectra (300 - 460 nm).

For standard CNA and DCNA calibration solutions, the excitation-emission maps in the range 300 - 460 nm x 360 - 550 nm with a step of 2 nm were also recorded. The measurements for pure methanol were carried out as well, in order to obtain a reference signal related to light scattering.

In order to determine the Stern-Volmer quenching constants, the following procedure was conducted for all single substances used to prepare a mixture: a constant 1.5 mL volume of the flourescent solution ($A \approx 0.1$) was taken into a cuvette and the fluorescence spectrum was measured. Next, the 0.1 mL portion of the 0.06 M potassium iodide solution in methanol was added and the spectrum was recorded again. The cycle was repeated several times, until the total concentration of the quencher in the sample was equal to 0.02 M.

4.3 Measurements of emission spectra for three-component mixtures

All measurements were performed practically in the same way as for solutions of single substances. A subtle differences referring to the recording of the quenched fluorescence spectra are noted below.

First, the portions of the quencher solutions added to the initial 1.5 mL of the sample were reduced to 0.08 mL. Also, the 'second type' of potassium iodide solution, that is KI dissolved in ternary mixture solution, was used. In consequence, the concentration of all three fluorophores was maintained constant during all the performed operations.

The spectra were recorded for five different excitation lines referring to the evolving level of the signal complexity: 425 nm (DCNA selective range), 400 nm (binary signal CNA+DCNA), 365, 355 and 345 nm (ternary signal A+CNA+DCNA).

The excitation-emission maps were recorded three times: for the initial sample of the mixture and after the addition of the third (twice the intensity drop) and seventh (fourfold drop) portion of the quencher.

5. Data pre-processing

The signal obtained for pure solvent was subtracted from the spectra and excitation-emission maps of all the measured samples. In some cases it was necessary to scale the background signal, so the signal associated with Rayleigh and Raman scattering was (at least partially) removed. In addition, the fluorescence and excitation spectra were smoothed using a Whittaker smoother [P.H.C. Eilers (2003) A perfect smoother, *Analytical. Chemistry* **75**: 631-636].

In the case of the individually quenched fluorescence spectra, the signal intensity was corrected for the fluorophore concentration change (dilution by addition of the KI solution in methanol). The correction was not necessary for analogous spectra of the mixture, as the added quencher was dissolved in the examined mixture solution (concentration of the fluorescent components was maintained constant). Both the 'raw' and pretreated data can be found attached in ***Appendix D*****.**

6. ***A few additional remarks***

When designing the ‘training’ model system, the proposed fluorophores may be successfully replaced by other substances of similar character, depending on their availability. However, it may be then necessary to change as well the applied quencher. For instance, it was established during the preliminary studies that quenching the fluorescence of 9-chloro-10-cyano-anthracene (CAS: 1213-82-7) with potassium iodide produces the Stern-Volmer quenching constant, K_{SV}, equal approximately to 160 M^{-1}. Since it is very close to the S-V constants obtained for CNA and DCNA, severe complications in the data analysis may occur (an actually do occur). Since it is not beneficial for the demonstrative experiment, the bromide salts (KBr and LiBr) can instead be used as quenchers [**39, 40**].

The second practical remark, based on observations, is that at least for demonstrative, educational reasons it is good to maintain a similar level of emission intensity of all the fluorophores in a mixture. As a supporting criterion, either the values of the fluorescence quantum yield or the fluorescence spectra of individual standard solutions can be used. By way of illustration; the signal obtained for the mixture, where ratio of the used CNA and DCNA bulk solution is 4:5 or higher (the manuscript suggests 5:3), is almost entirely dominated by the emission of DCNA. Consequently, the spectral dataset analysis may be then highly problematic and lead to wrong results, which is definitely undesired from a didactical point of view.

APPENDIX C

MATLAB codes

For all data matrices, denoted later as `<data_matrix>` (or `<data_vector>`), a column (vertical) 2D array is assumed – thus the fluorescence spectra are localised in consecutive columns. Associated sets of wavelengths are marked as `<wavelengths>`. Symbol `<SF_value>` refers to the number of principal components (or significant factors) responsible for the system total variance.

The code presented below is in a form of manually triggered commands, dedicated for educational purposes to a step-by-step analysis of the particular type of data. It can be, however, easily generalised for any type of a problem and transformed into a form of universal routines run automatically for the provided input data files (in MATLAB with an initial keyword `function`).

1. *PCA algorithm – determination of the number of significant factors on the example of excitation-emission maps (EEMs)*

```
>> PCA_MAP = <data_matrix>;  % data matrix (EEM) is defined
>> lambda_EM = <wavelengths>; % set of associated wavelengths
>> lambda_EX = <wavelengths>; % is defined
>>
>> [u, s, v] = svd(PCA_MAP, 0); % SVD procedure is called
>> s_values = diag(s); % singular values are extracted
>> % Statistical parameters are computed:
>> eig_val = s_values.^2; % eigenvalues
>> eig_sum = sum(eig_values); % summaric variance of the dataset
>> rel_variance = eig_values./eig_sum; % relative variance of each factor
>> for n = 1:size(eig_values,1)                 % total variance described
     tot_variance(n) = sum(rel_variance(1:n)); % by n factors
>> end
>>
>> f = <SF_value>; % on the basis of the above parameters, the number of
>>                 % significant factors should be determined
>>
>> % Verification with graphical criterion
>> f = 3; % example: three significant factors -> f = 3
>>
>> figure(1)
>> plot(lambda_EM, u(:,1:f+1))      % eigenvectors (abstract  emission spectra)
>> legend('u1', 'u2', 'u3', 'u4')   % are plotted for the first f+1 factors
>>
>> figure(2)
>> plot(lambda_EX, v(:,1:f+1))      % eigenvectors (abstract  excitation spectra)
>> legend('v1', 'v2', 'v3', 'v4')   % are plotted for the first f+1 factors
```

2. Data reproduction procedure – continuation of the PCA approach

```
>> f = <SF_value>; % the number of significant factors is defined
>> MAP_REP = u(:,1:f)*s(1:f,1:f)*v(:,1:f)'; % the data matrix MAP_PCA is
>>                                           % reproduced with the SVD matrices
>> NOISE = PCA_MAP - MAP_REP; % the error ('noisy') matrix is calculated
>>
>> figure(3)
>> surf(lambda_EX, lambda_EM, NOISE) % the cut off signal is plotted
>> figure(4)
>> plot(lambda_EM, PCA_MAP, 'k') % the reproduced and the original spectra
>> hold on                       % are graphically compared
>> plot(lambda_EM, PCA_REP, 'r')
```

3. TFA algorithm – validation of the mixture composition (EEM example)

```
>> MAP_REP = <data_matrix>;  % reproduced data matrix (EEM) is defined
>> S_TEST = <data_vector>;  % 'target' spectrum is defined
>> lambda_MAP = <wavelengths>; % set of wavelengths is defined
>> lambda_S = <wavelengths>;   % for both EEM map and 'target' spectrum
>>
>> S_TEST = spline(lambda_S, TEST_S, lambda_MAP); % spectral range of 'target'
>>                                                 % spectrum is adjusted to EEM
>> S_TEST = TEST_S./max(TEST_S); % 'target' spectrum is normalised to a unit
>>                               %  maximum
>> [u, s, v] = svd(MAP_REP, 0); % SVD procedure is called
>>
>> f = <SF_value>; % the number of significant factors is defined
>>
>> % Determination of linear combination coefficients for reproduction of
>> % the 'target' spectrum with eigenvectors u:
>> % Version 1 - projection approach (scalar product)
>> p_coef = u(:,1:f)'*S_TEST; % scalar product is calculated
>> % Version 2 - the least squares approach
>> p_coef = u(:,1:f)\S_TEST; % optimal coefficients are estimated
>>
>> S_CALC = u(:,1:f)*p_coef; % the 'target' spectrum is reproduced
>>
>> % Comparison of the original and reproduced 'target' spectrum
>> figure(1)
>> axis 'square' % x and y axis are set to equal length
>> line([0,1],[0,1]) % ideal one-to-one relationship (y = x)
>> hold on
>> plot(S_TEST, S_CALC, 'x') % real relationship between the two spectra
>>
>> figure(2) % graphical comparison of the spectra
>> plot(lambda_MAP, S_TEST, 'k') % original spectrum
>> hold on
>> plot(lambda_MAP, S_CALC, 'r') % projected (reproduced) spectrum
```

4. Iterative RAFA algorithm – determination of individual contributions of components to the mixture spectra (EEM example)

```
>> MAP_A_REP = <data_matrix>;  % reference data matrix is defined
>> MAP_B_REP = <data_matrix>;  % tested data matrix is defined
>>
>> tau = <data_vector>;    % the scanning range of tau parameter is defined
>> tau = (0:0.01:1.00)';  % example: tau = 0.00, 0.01, 0.02, …, 1.00
>>
>> for i = 1:size(tau,1) % the beginning of the iterative loop: for each tau...
>>    D_MX = MAP_B_REP - tau(i)*MAP_A_REP; % ...calculate the difference matrix
>>    [u, s, v] = svd(D_MX, 0); % ...call SVD to allow its decomposition
>>    s_values(i,:) = diag(s); % ...and extract the resulting singular values
>> end % the end of the cycle
>>
>> f = <SF_value>; % the number of significant factors is defined
>>
>> plot(tau, log(s_values(:,f))) % the evolution of the 'last' significant
>>                               % singular value is plotted against tau
>>
>>  tau0 = [<data_vector>] % tau values corresponding to the local (global)
>>                         % minima read manually from the plot
>>
>>
>> % Automation of the procedure - search for the minimum with differential
>> % sign change criterion
>>
>> dif_eig_val = diff(eig_val(:,f)); % differential is calculated numerically
>> tau0 = 0;  % allocation of output
>> % iterative cycle: a) for each tau b) check if the sign of the differential
>> % changes from - to +; c) YES -> note down this tau value, as it refers to
>> % a local minimum;

>> for i = 2:size(dif_eig_val,1) % <- step a
>>    if (dif_eig_val(i-1) <= 0) & (dif_eig_val(i) > 0) % <- step b
>>        tau0 = [tau0; tau(i)]; % <- step c
>>    end
>> end
>>
>> tau0 = tau0(2:end); % delete the allocated zero value
```

5. EFA algorithm – estimation of the complexity (variability) evolution among the experimental dataset (example for EEM)

```
>> MAP_REP = <data_matrix>;  % reproduced data matrix (EEM) is defined
>> lambda_EM = <wavelengths>; % set of associated wavelengths
>> lambda_EX = <wavelengths>; % is defined
>>
>> f = <SF_value>; % the total number of significant factors is defined
>>
>> % EFA procedure: for the particular spectral range n: a) define the
>> associated data submatrix, b) decompose it with SVD and c) extract the
>> resulting singular values; repeat until subrange n is equal to full range N
>>
>> % EVOLVING NUMBER OF ROWS (emission wavelength mode), FORWARD DIRECTION
>> for n = f : 1 : size(MAP_REP,1) % f - > N direction, interval: +1;
>>    M_MX = MAP_REP(1:n,:); % <- step (a
>>    [u, s, v] = svd(M_MX, 0); % <- step (b
>>    EFA_R_F(n,:) = diag(s(1:f,1:f)); % <- step (c
>> end
>>
>> % EVOLVING NUMBER OF ROWS (emission wavelength mode), BACKWARD DIRECTION
>> for n = size(MAP_REP,1)-f : -1 : 1 % N - > f direction,  interval: -1;
>>    M_MX = MAP_REP(n:end,:);
>>    [u, s, v] = svd(M_MX, 0);
>>    EFA_R_B(n,:) = diag(s(1:f,1:f));
>> end
>>
>> % EVOLVING NUMBER OF COLUMNS (excitation wavelength mode), FORWARD DIRECTION
>> for n = f : 1 : size(MAP_REP,2) % change of array rows -> columns
>>    M_MX = MAP_REP(:,1:n);
>>    [u, s, v] = svd(M_MX, 0);
>>    EFA_C_F(n,:) = diag(s(1:f,1:f));
>> end
>>
>> % EVOLVING NUMBER OF COLUMNS (excitation wavelength mode), BACKWARD DIRECTION
>> for n = size(MAP_REP,2)-f : -1 : 1
>>    M_MX = MAP_REP(n:end,:);
>>    [u, s, v] = svd(M_MX, 0);
>>    EFA_C_B(n,:) = diag(s(1:f,1:f));
>> end
>>
>> %  As the initial submatrix consists of f vectors, the sets of obtained
>> %  singular values have to be corrected for the 'skipped' f-1 values
>> %  (iteration should begin from 1, not from f)

>> for n = 1:f-1 % f-1 first or f-1 final rows remain undefined
>>    EFA_C_B = [EFA_C_B; EFA_C_B(size(EFA_C_B,1),:)];  % missing rows are
>>    EFA_R_B = [EFA_R_B; EFA_R_B(size(EFA_R_B,1),:)];  % filled in with copies
>>    EFA_C_F(n,:) = EFA_C_F(f,:);                      % of the nearest fully
>>    EFA_R_F(n,:) = EFA_R_F(f,:);                      % defined vector
>> end
```

```
>> % Generation of the EFA plots - combining 'forward' and 'backward' evolution
>> % of singular values (and choosing from them the minimal value).
>> Z = EFA_C_B > EFA_C_F; % A logical array Z is defined...
>> EFA_C_PLOT =  EFA_C_F.*Z +  EFA_C_B*(1-Z); % ... which is then used to choose
>> Z = EFA_R_B > EFA_R_F;                      % the minimal value
>> EFA_R_PLOT =  EFA_R_F.*Z +  EFA_R_B*(1-Z);
>>
>> figure(1) % The resulting graph is made (a version without normalisation)
>> for i = 1:f
>>    subplot(1,2,1) % subplot for the excitation wavelength set
>>    hold on
>>    area(lambda_EX, EFA_C(:,i))
>>    subplot(1,2,2) % subplot for the emission wavelength set
>>    hold on
>>    area(lambda_EM, EFA_R(:,i))
>> end
```

6. *Non-iterative GRAM algorithm (the most basic version) – simultaneous determination of contributions to the mixture matrix from a few components (qualitative and/or quantitative analysis)*

```
>> MAP_A_REP = <data_matrix>;  % reference data matrix is defined
>> MAP_B_REP = <data_matrix>;  % tested data matrix is defined
>> lambda_EM = <wavelengths>; % set of associated wavelengths
>> lambda_EX = <wavelengths>; % is defined
>>
>> f_A = <SF_value>; % the number of significant factors is defined
>> f_B = <SF_value>; % for both matrices
>> f = max(f_A, f_B); % the bigger value of f is chosen
>>
>> if  f_A == f
>>     MAP_MAX = MAP_A_REP;   % the more complex data matrix
>>     MAP_MIN = MAP_B_REP;   % is to be decomposed
>> else
>>     MAP_MAX = MAP_B_REP;
>>     MAP_MIN = MAP_A_REP;
>> end
>>
>> [u, s, v] = svd(MAP_A_REP, 0); % SVD procedure is called
>>
>> H_MX = u(:,1:f)'*MAP_B_REP*v(:,1:f)/s(1:f,1:f); % an auxiliary matrix H
>>                                                 % is created
>>
>> [eig_vec, eig_val] = eig(H_MX); % eigenvalue-eigenvector problem
>>                                 % is solved for H
```

```
>> tau0 = diag(eig_val); % the contribution (concentration) ratios are equal to
>>                        % eigenvalues (tau0 = C_B / C_A, fA >= fB)
>>
>> EM_spectra =  u(:,1:f)*eig_vec;                    % individual emission and
>> EX_spectra =  inv(eig_vec)*s(1:f,1:f)*v(:,1:3)'; % excitation spectra are
>> EX_spectra = EX_spectra';                          % retrieved for components
>>
>> Since spectra estimated by GRAM may be fully negative, their sign should be
>> checked and eventually the additive inverse should be calculated
>>
>> for i = 1:f  % validation for all f obtained spectra
>>    if abs(max(EX_spectra(:,i))) < abs(min(EX_spectra(:,i))) % the comparison
>>       EM_spectra(:,i) = -EM_spectra(:,i);                    % of absolute and
>>    end                                                       % minimal/maximal
>>                                                              % value is used
>>    if abs(max(EX_spectra(:,i))) < abs(min(EX_spectra(:,i))) % as a criterion
>>       EX_spectra(:,i) = -EX_spectra(:,i);                    % of full
>>    end                                                       % negativity
>> end
>>
>> % The spectra are presented in a graphical form
>> figure(1)
>> subplot(1,2,1)
>> plot(lambda_EX, EX_spectra)
>> subplot(1,2,2)
>> plot(lambda_EM, EM_spectra)
```

7. ALS algorithm of the spectra refinement – on the example of the results previously obtained from GRAM with implemented non-negativity constraint

```
>> for i = 1:50 % number of the refinement cycles
>>
>> Z = EM_spectra > 0 % logical matrix of nonnegativity constraint(+ = 1, - = 0)
>> EM_spectra =  EM_spectra.*Z; % negative values are zeroed (emission spectra)
>>
>> EX_spectra = EM_spectra\MAP_A_REP % 'new' (refined) excitation spectra are
>>                                   % calculated
>>
>> Z = EX_spectra > 0 % creation of the logical matrix (excitation spectra)
>> EX_spectra =  EX_spectra.*Z; % negative values are zeroed
>>
>> EM_spectra =  MAP_A_REP/EX_spectra; % 'new' (refined) emission spectra are
>>                                     % calculated
>> end % the end of ALS cycle
```

8. GRAM algorithm applied for determination of the Stern-Volmer quenching constants

```
>> Q_SET_EX1 = <data_matrix>; % three reproduced matrices of quenched
>> Q_SET_EX2 = <data_matrix>; % fluorescence, recorded at three different
>> Q_SET_EX3 = <data_matrix>; % excitation lines, are defined
>> Q = <data_vector>; % quencher concentrations in ascending order
>>                    % (0; q1; q2 ; …) are defined
>>
>> f = <SF_value>; % the number of significant factors is defined
>>
>> % Reference matrix is constructed from the spectra recorded
>> % for the unquenched fluorescence
>> MX_0 = [Q_SET_EX1(:,1), Q_SET_EX2(:,1), Q_SET_EX3(:,1)];
>> [u, s, v] = svd(MX_0, 0); % SVD procedure is called for the reference matrix
>>
>> for q = 2:size(Q,1) % iterative loop: for each quencher concentration q ...
>>
>>    % ...form a dataset matrix to be compared with that obtained
>>    % for the unquenched sample
>>    MX_Q = [Q_SET_EX1(:,q), Q_SET_EX2(:,q), Q_SET_EX3(:,q)]; macierz MX_Q dla
>>
>>    % ... and then apply the full GRAM algorithm, that is
>>    H_MX = u(:,1:f)'*MX_Q*v(:,1:f)/s(1:f,1:f); % construct an auxiliary
>>    % matrix H and solve the resulting eigenvector-eigenvalue problem
>>    [eig_val] = eig(H_MX);
>>    IQ_I0_ratio(i,:) = sort(eig_val,'descend'); % (extract eigenvalues
>>                                                % in descending order);
>>
>> end % the end of the iterative loop
>>
>> IQ_I0_ratio(1,:) = 1; % value for q = 0 is defined as a unit (I0 / I0 = 1)
>>
>> SV_plot =  1./IQ_I0_ratio; % the proper intensity ratio defined by the Stern-
>>                            % Volmer equation is calculated (I0/IQ= 1 + KSV*Q)
>> KSV =  Q\(SV_plot - 1); % Stern-Volmer quenching constants are determined
>>
>> % In order to assign the KSV values to proper fluorophores, the individual
>> % fluorescence spectra may be retrieved as:
>> H_MX = u(:,1:f)'*MX_Q*v(:,1:f)/s(1:f,1:f); % (a supplementary matrix H)
>> [eig_vec, eig_val] = eig(H_MX); % (eigenproblem solution)
>> EM_spectra =  u(:,1:f)*eig_vec; % retrieving the spectra of pure components
```

9. 'Cascade' RAFA algorithm applied for extracting a series of the fluorescence spectra of individual components from the spectra of the quenched fluorescence of a mixture

```
>> Q_425nm = <data_matrix>;  % three reproduced matrices of quenched
>> Q_400nm = <data_matrix>;  % fluorescence, recorded at three chosen
>> Q_355nm = <data_matrix>;  % excitation lines, are defined
>>
>> Q_DCNA = Q_425nm; % signal for one of the datasets is selective (DCNA)
>> tau = (0.0:0.1:3.0)' % the scanning range of tau parameter is defined
>>
>> % the RAFA cycle for the spectra of a two-component mixture (CNA+DCNA)
>> % is commenced;
>> for i = 1:size(tau,1) % RAFA loop: for each tau...
>>    D_MX = Q_400nm - tau(i)*Q_DCNA; % ...calculate the difference matrix
>>    [u, s, v] = svd(D_MX, 0); %...call SVD for its decomposition
>>    s_val(i,:) = diag(s); % ..and extract the resulting singular values
>> end % the end of the cycle
>>
>> plot(tau, log(s_val(:,2))) % graphical validation is performed to confirm if
>> % the global minimum of the 2nd singular value was obtained
>> i_opt = find(s_val(:,2) == min(s_val(:,2))); % the value of tau at the
>> tau0 =  tau(i_opt);                          % minimum is read from the plot
>>
>> Q_CNA = Q_400nm - tau0*Q_DCNA; % binary signal is purified from contribution
>>                                % of DCNA, 'pure' spectra of CNA are obtained
>>
>> % The first RAFA cycle for the spectra of a ternary mixture (A+CNA+DCNA)
>> % is initiated;
>> for i = 1:size(tau,1)                  % Contribution of DCNA to the mixture
>>    D_MX = Q_355nm - tau(i)*Q_DCNA;     % signal is determined if the minimum
>>    [u, s, v] = svd(D_MX, 0);           % of the third singular value is found
>>    s_val(i,:) = diag(s);
>> end
>> plot(tau, log(s_val(:,3))) % evolution of the third singular value is plotted
>> i_opt = find(s_val(:,3) == min(s_val(:,3))); % after validation, tau value
>> tau0 =  tau(i_opt);                          % at the minimum is saved
>>
>> Q_355nm_RED = Q_355nm - tau0*Q_DCNA; % the DCNA contribution is subtracted
>>                                      % from the ternary signal
>>
>> % The second RAFA cycle for the reduced dataset (A+CNA) is  performed;
>> for i = 1:size(tau,1)                  % Contribution of CNA to the spectra
>>    D_MX = Q_355nm_RED - tau(i)*Q_CNA;  % is determined; the minimum of the
>>    [u, s, v] = svd(D_MX, 0);           % second singular value is captured
>>    s_val(i,:) = diag(s);
>> end
>>
>> plot(tau, log(s_val(:,2))) % evolution of the second singular value
>>                            % is plotted
>> i_opt = find(s_val(:,2) == min(s_val(:,2))); % optimal tau value
>> tau0 =  tau(i_opt);                          % is defined
>> Q_A = Q_355nm_RED - tau0*Q_CNA; % binary signal is purified from contribution
                                   % of CNA, 'pure' spectra of A are obtained
```
